# Achieving cell-type specific transduction with adeno-associated viral vectors in pigeons

**DOI:** 10.1016/j.crneur.2026.100157

**Published:** 2026-04-03

**Authors:** John Michael Tuff, Kevin Haselhuhn, Tatjana Surdin, Stefan Herlitze, Marie Ziegler, Onur Güntürkün, Noemi Rook

**Affiliations:** a Department of Biopsychology, Institute of Cognitive Neuroscience, Faculty of Psychology, Ruhr University Bochum, Universitätsstraße 150, Bochum, 44801, Germany; b Max Planck School of Cognition, Stephanstraße 1a, Leipzig, 04103, Germany; c Department of General Zoology and Neurobiology, Ruhr University Bochum, Universitätsstraße 150, Bochum, 44801, Germany; d Research Center One Health, Ruhr University Bochum, Universitätsstraße 150, Bochum, 44801, Germany

## Abstract

Birds are valuable models for studying learning, cognition, song, and vision, yet tools for controlling and recording brain activity with millisecond precision remain underutilized in avian research. Advances in methods such as chemogenetics, optogenetics, and in vivo imaging have transformed rodent studies but require gene delivery techniques, like adeno-associated viruses (AAVs), in non-transgenic species. This study validates AAV tools for precise gene expression in pigeons. We identified both AAV1 and AAV8 as useable vectors, demonstrating strong neuronal tropism and anterograde/retrograde transgene expression, while AAVretro was ineffective. The CaMKIIα promoter and mDLX enhancer enabled cell-type-specific expression, targeting predominantly excitatory and inhibitory neurons, respectively. Additionally, we established proof of concept for the expression of NpHR (a chloride pump) and demonstrated the functionality of conditional gene expression systems using the Cre-loxP system. These advancements expand the genetic toolkit for pigeons, facilitating precise manipulation of neural circuits and enabling future studies on complex avian behaviors and brain functions. By bridging molecular tools and avian neuroscience, this work paves the way for comparative and translational research, offering insights into the neural basis of cognition and sensory processing in birds.

## Highlights

• AAV8 enables efficient and non-toxic neuronal gene delivery in pigeons.
• CaMKIIα and mDLX systems drive cell-type-preferential gene expression.
• Cre/loxP system validated for conditional expression.
• AAVretro shows limited utility for retrograde labeling in pigeons.
• Expands the genetic toolkit for avian neuroscience applications.

## Graphical abstract

**Figure:**
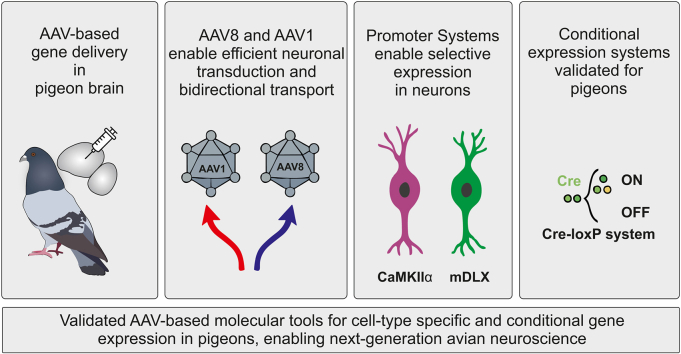

## 1 Introduction

Avian species have emerged as valuable models in neuroscience research due to their diverse cognitive abilities and specialized behaviors. For instance, crows display cognitive skills comparable to those of primates (Balakhonov and Rose, 2017; Emery and Clayton, 2004; Nieder, 2017), while zebra finches are widely used in studies of vocal learning, offering insights relevant to human language (Brainard and Doupe, 2002; Jarvis, 2004; Steinemer et al., 2024). Pigeons, on the other hand, are renowned for their exceptional visual processing and navigational abilities (Biro et al., 2007; Rook et al., 2021; Wallraff, 2001). Despite the distinct organizational differences between avian and mammalian brains (Güntürkün and Bugnyar, 2016), recent research suggests that certain principles of sensory system organization are conserved across species (Stacho et al., 2020). This growing body of evidence highlights the value of comparative studies, which offer critical insights into how brain functions emerge from neural structures in both birds and mammals (Brenowitz and Zakon, 2015).

Especially with the emergence of groundbreaking techniques like optogenetics, which enable precise control over neuronal activity, the possibilities for advancing neuroscientific research are rapidly expanding. Optogenetics can be used to control and manipulate neuronal activity using genetically modified light-sensitive proteins, such as ion channels (e.g. channelrhodopsin) or chloride pumps (e.g. halorhodopsin) with millisecond accuracy and cell-type specificity, which cannot be achieved with traditional manipulation methods such as pharmacology or electrical stimulation (Li et al., 2005; Aravanis et al., 2007). Through its growing popularity, optogenetics has been established in a wide variety of model species, most prominently in mammals, including rodents (Galvan et al., 2018), primates (Han et al., 2009) and ferrets (Zhou et al., 2016), but also in birds, such as zebra finches (Roberts et al., 2012) and pigeons (Rook et al., 2021). In contrast to rodents, transgenic bird models are not yet available, making gene transfer methods essential for the successful application of optogenetics in avian species. A widely used approach is viral gene transfer, which employs vectors such as adeno-associated viruses (AAVs) or lentiviruses. These vectors can be administered locally or systemically (Emiliani et al., 2022), delivering genetic material encoding the selected opsin. Once inside the target cells, the genetic material is expressed, allowing the opsin to integrate into the cell membrane. When selecting a viral vector, it is essential to consider several factors, including the type of virus, the appropriate serotype, and the choice of promoter system (Emiliani et al., 2022). In the context of optogenetics, adeno-associated viruses (AAVs) are commonly used, often with AAV2 genomes pseudotyped with various AAV capsids to enhance targeting specificity. The efficiency of viral vectors is highly dependent on the viral serotype, which can vary significantly across different tissues and species (Lisowski et al., 2015; Wu et al., 2006). For instance, a recent study in pigeons compared the transduction efficiencies of AAV1, AAV5, and AAV9. The results showed that AAV1 reliably achieved robust transgene expression, AAV5 produced virtually no expression, and AAV9 had neurotoxic effects (Rook et al., 2021). Another key consideration in selecting an appropriate viral vector is choosing the right promoter system to target specific neuronal populations (Emiliani et al., 2022). Promoters are included in the gene sequence within the viral vector and are vital for opsin expression. Different promoters are for example able to drive opsin expression in either all cell types (e.g. CAG) or specifically in certain cell types (e.g. CaMKIIα for excitatory cells or mDLX for GABAergic cells) (Han et al., 2009; Dimidschstein et al., 2016). In pigeons, behavioral manipulations to date have exclusively utilized the widely used synthetic CAG promoter, which is driving robust and ubiquitous gene expression in a variety of cell types and organisms (Rook et al., 2021). To advance future studies, it is essential to demonstrate that other promoter systems can reliably drive gene expression with comparable specificity to that observed in mammals. These complexities highlight the need for dedicated investigations in species where the properties of viral vectors remain less well understood. In this study, we evaluate the efficiency of various viral vectors in transducing different regions of the pigeon brain. Building on previous findings that identified AAV1 as the most effective construct in pigeons, we compared its performance to AAV8, a serotype known for reliable expression in other species. Furthermore, we examined the efficacy of several promoter systems, including CaMKIIα for targeting CaMKIIα-positive cells, mDLX for GABAergic cells, as well as the Cre-loxP system for conditional expression.

## 2 Results

### 2.1 Assessment of AAV8 for gene transfer in pigeon brains

**Fig. 1:**
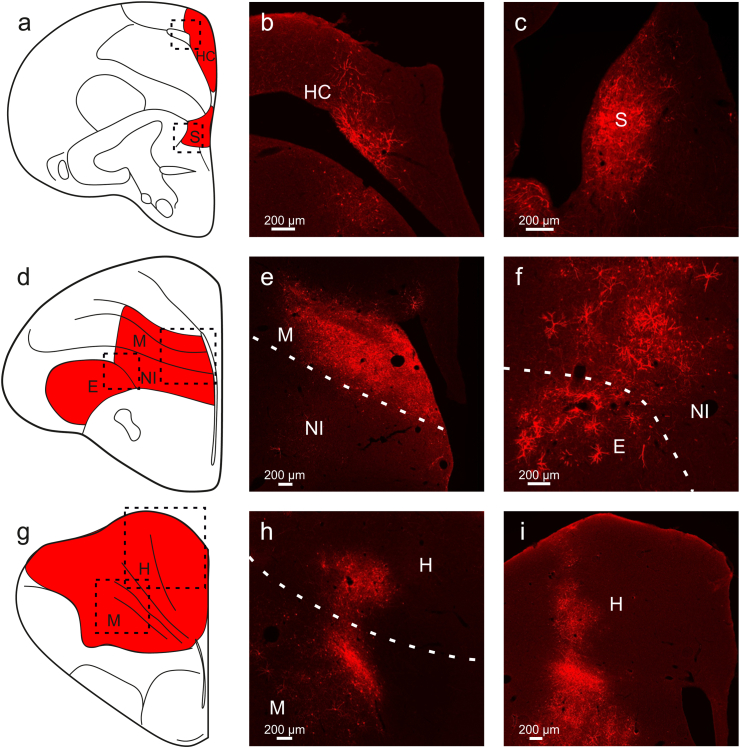
**Transgene expression after AAV8 injection into various regions of the pigeon brain** (a, d, g) Schematic illustration of pigeon brain regions where expression was observable after injections of AAV8-CAG-tdTomato. Injections were performed in five hemispheres in three animals. Expression was found in the (b) hippocampus, (c) septum, (e, h) mesopallium, (f) nidopallium, (f) entopallium, and (h, i) hyperpallium. All scale bars represent 200 μm.

**Fig. 2:**
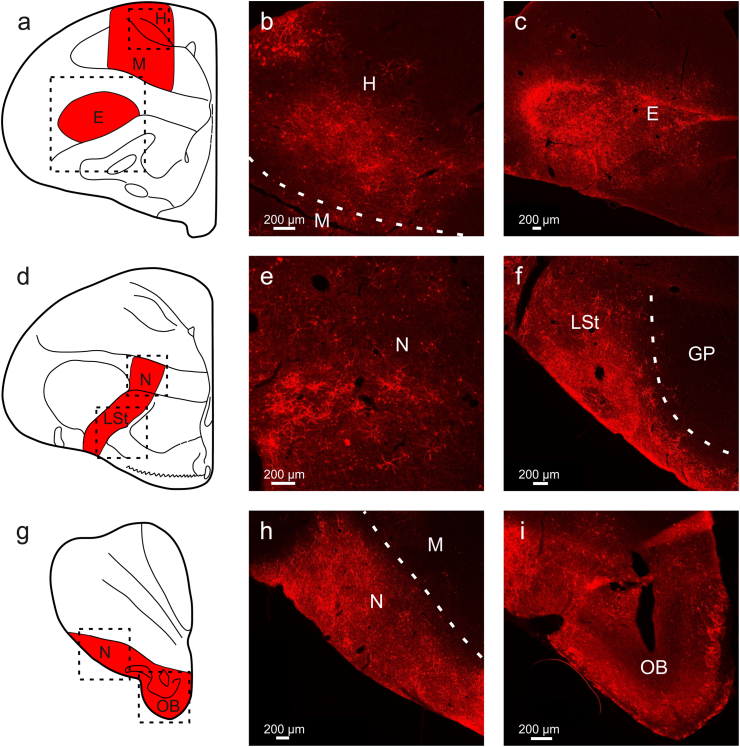
**Transgene expression after AAV1 injection into various regions of the pigeon brain** (a, d, g) Schematic illustration of pigeon brain regions where expression was observable after injections of AAV1-CAG-tdTomato. Injections were performed in five hemispheres in three animals. Expression was found in the (b) hyperpallium, (c) entopallium, (e, h) nidopallium, (f) lateral striatum, and (i) olfactory bulb. All scale bars represent 200 μm.

Previous studies have highlighted significant differences in the efficiency of adeno-associated virus (AAV) serotypes in transducing cells in avian brains (Rook et al., 2021; Nimpf et al., 2024). For example, Rook et al. (2021) found that AAV1 is highly effective, while AAV5 and AAV9 result in little to no expression (Rook et al., 2021). To expand the existing toolkit, we decided to investigate the suitability of AAV8, as previous studies in rodents reported a transduction preference for non-neuronal cells (Castle et al., 2016; Aschauer et al., 2013). We injected AAV8 into various brain regions and observed widespread expression in areas such as the hippocampus (Fig. 1a and b), septum (Fig. 1a and c), mesopallium (Fig. 1d,e,g,h), nidopallium (Fig. 1d and f), entopallium (Fig. 1d and f), and hyperpallium (Fig. 1g,h,i). We also injected AAV1-CAG-tdTomato for a better comparison to AAV8 and to replicate prior findings for this serotype. We found expression in the hyperpallium (Fig. 2a and b), entopallium (Fig. 2a and c), nidopallium (Fig. 2d,e,g,h), lateral striatum (Fig. 2d and f) and olfactory bulb (Fig. 2g and i).

**Fig. 3:**
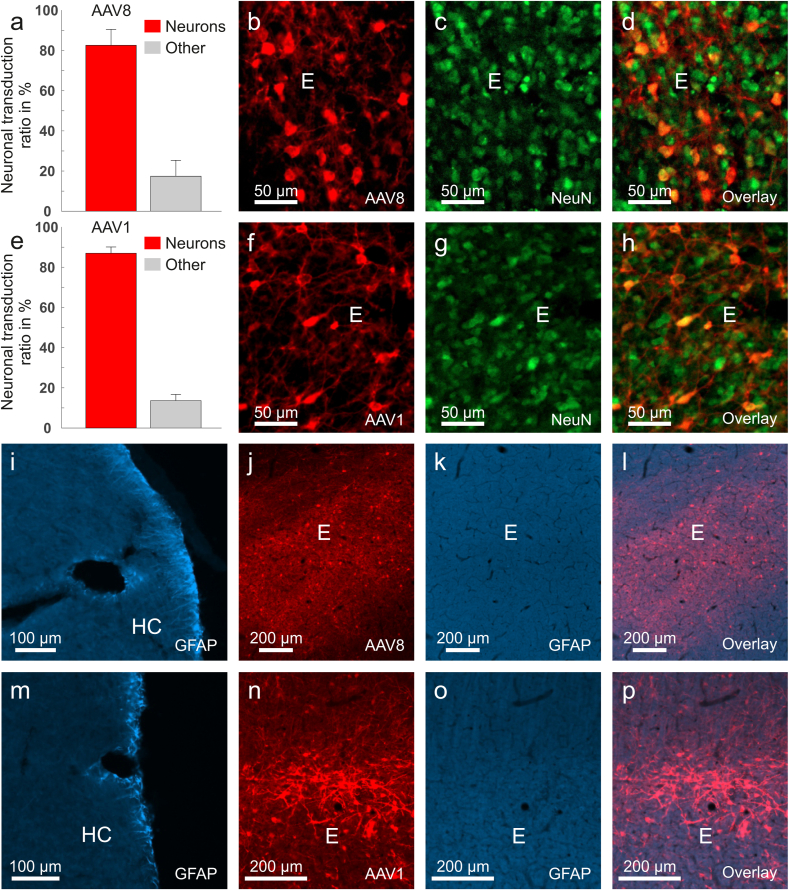
**Neuronal specificity and neurotoxicity for AAV1 and AAV8.** (a) Neuronal transduction ratio observed for injections with AAV8-CAG. (b-d) Fluorescence images depicting (b) RFP expression, (c) NeuN expression and (d) co-localization of RFP and NeuN for AAV8. (e) Neuronal transduction ratio observed for injections with AAV1-CAG (f-h)Fluorescence images depicting (f) RFP expression, (g) NeuN expression and (h) co-localization of RFP and NeuN for AAV1. Fluorescence images depicting (i, m) GFAP expression in a positive control area (the hippocampus), (j, n) RFP expression, (k,o) an absence of GFAP in the injection site and (l, p) co-localization of RFP and GFAP for (j-l) AAV8 and (n-p) AAV1. Injections were performed in five hemispheres of three pigeons. The neuronal transduction ratio was determined in a total of 22 sections for AAV8 and 19 sections for AAV1. Scale bars represent (b-d, f-h) 50 μm, (i, m) 100 μm or (j-l, n-p) 200 μm.

Given previous reports of AAV8's preference for non-neuronal cells (Castle et al., 2016; Aschauer et al., 2013), we performed double-labeling for RFP and NeuN, a neuronal marker, to determine the proportion of transduced neurons. Quantification of 22 brain slices from three animals, conducted independently by two examiners, revealed that 82.07% ± 6.53 SD of transduced cells co-localized with NeuN, confirming they were neurons (Fig. 3a–d). We also assessed the proportion of neuronal cells for AAV1 in 19 sections of three animals. For this serotype, we found 86.45% ± 3.10 SD of transduced cells to be co-localized with NeuN (Fig. 3e–h). To assess potential neurotoxicity, we examined GFAP expression. Neither AAV8 nor AAV1 induced GFAP expression at the injection sites (Fig. 3j–l,n-p). Notably, GFAP staining was visible in the hippocampus (Fig. 3i and m), a region where GFAP expression occurs naturally, confirming the staining's validity. These findings indicate that both AAV1 and AAV8 can drive transgene expression and are non-toxic for neuronal gene transfer in pigeons.

### 2.2 Comparison of AAV serotypes for anterograde and retrograde transport

**Fig. 4:**
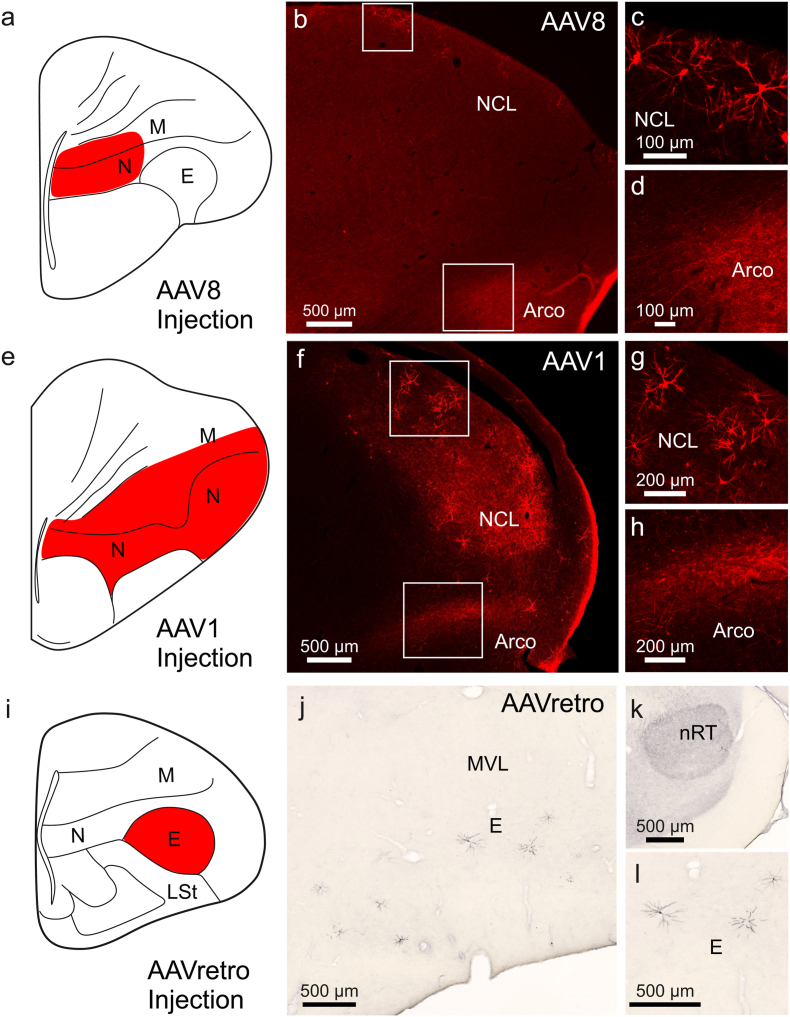
**Anterograde and retrograde properties of AAV1, AAV8 and AAVretro.** (a) Schematic image indicating the transduced area at the injection site of AAV8 in the medial nidopallium. (b) RFP-labeled fibers in the arcopallium and cell bodies in the NCL observed outside the injection site, indicating successful anterograde and retrograde expression of the transgene. (c) Higher magnification image of b. RFP-positive cells identified in the NCL, demonstrating retrograde transgene expression. (d) Higher magnification image of b. RFP-labeled fibers in the arcopallium, demonstrating anterograde transgene expression. (e) Schematic image indicating the transduced area at the injection site for AAV1 in the anterior nidopallium. (f) RFP-labeled fibers in the arcopallium and cell bodies in the NCL observed outside the injection site, indicating successful anterograde and retrograde expression of the transgene. (g) Higher magnification image of f. RFP-positive cells identified in the NCL, demonstrating retrograde transgene expression. (h) Higher magnification image of f. RFP-labeled fibers in the arcopallium, demonstrating anterograde transgene expression. (i) Schematic image indicating the transduced area at the injection site for AAVretro in the entopallium. (j) Representative image of ChR2 expression at the injection site following AAVretro delivery, highlighting local transduction efficiency. No ChR2-labeled fibers were observed outside the injection site, indicating unsuccessful anterograde transgene expression. (k) Virtually no ChR2-positive cells could be identified in regions distal to the injection site such as the nucleus rotundus, demonstrating virtually no retrograde transgene expression. The rounded area displaying a darker background corresponds to the nucleus rotundus, a structure characterized by naturally high signal intensity in DAB staining protocols. This is not indicative of non-specific labeling or an experimental artifact. (l) Higher magnification image of j depicting the injection site. The injected constructs were (a-d) AAV8-CAG-tdTomato (five hemispheres in three animals), (e-h) AAV1-CAG-tdTomato (five hemispheres in three animals) and (i-l) AAVretro (six hemispheres in three animals). Scale bars indicate (b, f, j, k, l) 500 μm, (c, d) 100 μm or (g, h) 200 μm.

Anterograde and retrograde transport are crucial features for applications such as projection-specific optogenetics. Previous studies have shown that different AAV serotypes exhibit varying degrees of transport capability. For instance, AAV1 demonstrates robust anterograde and retrograde transgene expression in pigeons (Rook et al., 2021). Additionally, specialized variants like AAVretro have been engineered to enhance retrograde transport efficiency (Tervo et al., 2016). To evaluate these properties, we compared the performance of AAV8 (Fig. 4a–d), AAV1 (Fig. 4e–h) and AAVretro (Fig. 4i–l). Consistent with previous findings, AAV1 showed strong anterograde transgene expression, evidenced by fiber expression in the arcopallium (Fig. 4f and h), and extensive retrograde labeling in cell bodies in the NCL (Fig. 4f and g) following injection into the anterior nidopallium (Fig. 4e). AAV8 also demonstrated significant anterograde transgene expression, with labeled fibers observed in the arcopallium (Fig. 4b and d), as well as retrograde labeling in the NCL (Fig. 4b and c) following injections into the anterior mesopallium/nidopallium (Fig. 4a). In contrast, AAVretro exhibited minimal expression, with signal largely confined to the injection site, the entopallium (Fig. 4i,j,l). Retrograde labeling was virtually absent, with only sparse dendritic labeling detected in the nucleus rotundus following entopallium injections(Fig. 4 k). This projection was chosen for AAVretro as prior work demonstrated strong retrograde transport between these structures for AAV1 (Rook et al., 2021). These results suggest that AAVretro has limited utility in pigeons, while AAV8 and AAV1 are more effective for both anterograde and retrograde transport.

### 2.3 Validation of cell-type specific promoter/enhancer systems for transgene expression

**Fig. 5:**
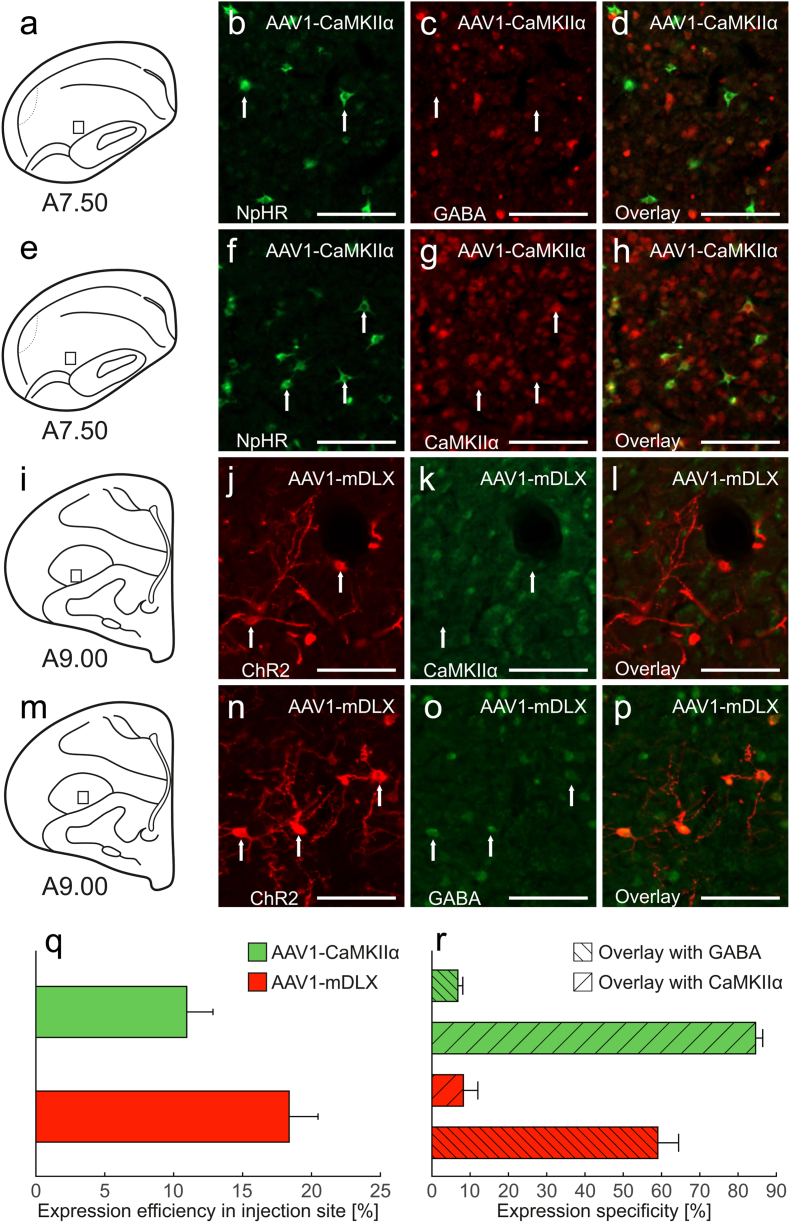
**CaMKIIα and GABA specificity of CaMKIIα and mDLX promoter/enhancer systems.** Fluorescence images of sections after injection of either (a-h) AAV1-CaMKIIα-eNpHR3.0-EYFP (n = eight hemispheres in five animals) or (i-p) AAV1-mDLX-ChR2-mCherry (n = six hemispheres in four animals). (a) Schematic illustration indicating the location of AAV1-CaMKIIα injection. (b) Expression of NpHR after virus injection. (c) Fluorescence staining against GABA. (d) Overlay of NpHR and GABA signals showing no co-localizations. (e) Schematic illustration indicating the location of AAV1-CaMKIIα injection. (f) Expression of NpHR after virus injection. (g) Fluorescence staining again CaMKIIα. (h) Overlay of NpHR and CaMKIIα showing co-localization of both signals. (i) Schematic illustration indicating the location of AAV1-mDLX injection. (j) Expression of ChR2 after virus injection. (k) Fluorescence staining against CaMKIIα. (l) Overlay of CaMKIIα and ChR2 signals showing no co-localization. (m) Schematic illustration indicating the location of AAV1-mDLX injection. (n) Expression of ChR2 after virus injection. (o) Fluorescence staining against GABA. (p) Overlay of ChR2 and GABA showing co-localization of both signals. (q) Expression efficiency of AAV1-CaMKIIα and AAV1-mDLX in the injection site. (r) Expression specificity for both constructs with GABA and CaMKIIα. Transgene expression was visualized with counterstainings against (b-d, f-h) YFP or (j-l, n-p) RFP. All scale bars represent 100 μm.

We evaluated the specificity and usability of cell-type-specific promoter/enhancer systems for driving transgene expression in excitatory and inhibitory neurons. To this end, we paired AAV1 with either the CaMKIIα promoter, targeting CaMKIIα-positive excitatory neurons, or the mDLX enhancer, designed to drive expression in inhibitory interneurons (Kaur and Berg, 2022; Spool et al., 2021). To confirm specificity, brain sections from each vector were double-stained for CaMKIIα and GABA. For AAV1-CaMKIIα-NpHR, we observed strong transgene expression in several regions, including the entopallium, nidopallium, wulst, hippocampus, striatum and globus pallidus. Compared to the ubiquitous CAG promoter, expression levels were slightly lower, as expected for a cell-type-specific approach. Nevertheless, we found that 10.95 % ± 1.90 SEM CaMKIIα-positive neurons co-expressed the transgene (Fig. 5q). To determine the specificity of the CaMKIIα promoter, we determined the co-localization of transgene positive cells with either CaMKIIα or GABA. The co-localization analysis revealed a strong overlap with CaMKIIα-positive cells (84.57 % ± 1.87 SEM), with minimal overlap with GABA-positive cells (6.79 % ± 1.28 SEM) (Fig. 5a–h,r). While not entirely exclusive to CaMKIIα-positive neurons, the system exhibited a strong preference for this cell type. In contrast, for AAV1-mDLX-ChR2 we observed that 18.38 % ± 2.10 SEM of GABA-positive neurons were transgene-positive (Fig. 5q). Apart from that, transgene expression was predominantly localized to axons and dendrites in the entopallium, nidopallium and striatum. Co-localization analysis showed minimal overlap with CaMKIIα (8.22 % ± 3.78 SEM), while overlap with GABA-positive cells was higher (59.04 % ± 5.42 SEM) (Fig. 5i–p,r). These results suggest that while neither system achieves complete cell-type specificity, both exhibit clear preferences for their respective target cell populations.

### 2.4 Evaluation of conditional and advanced expression systems in pigeons

**Fig. 6:**
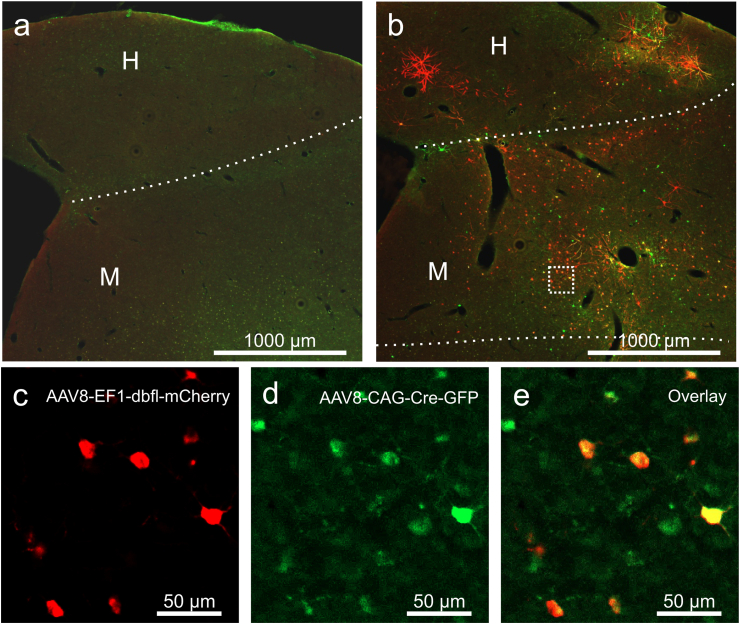
**Conditional transgene expression using the Cre-loxP system.** (a) No transgene expression can be seen when AAV8-EF1-dbfl-mCherry is injected without the presence of Cre. (b) Simultaneous injection of AAV8-EF1-dbfl-mCherry and AAV8-CAG-Cre-GFP leads to expression of GFP (green) and mCherry (red). (c) Close-up image of mCherry expression. (d) Close-up image of GFP expression. (e) Co-localization of mCherry and GFP. Injections of AAV8-EF1-dbfl-mCherry were performed in four hemispheres in two animals, and AAV8-CAG-Cre-P2A-eGFP was injected in two hemispheres in two animals. Scale bars represent (a, b) 1000 μm or (c, d, e) 50 μm.

**Fig. 7:**
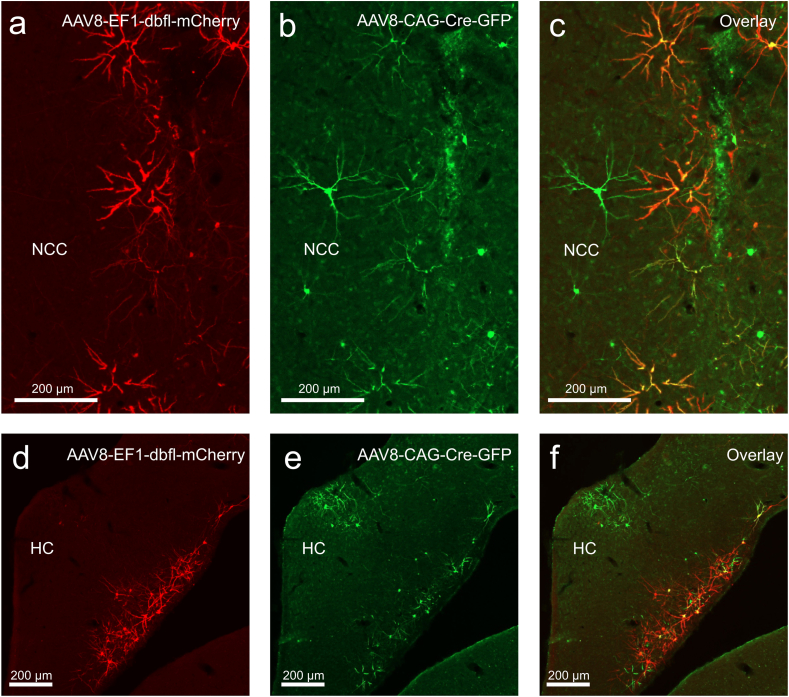
**Conditional transgene expression using the Cre-LoxP system.** (a) mCherry expression in the central caudal nidopallium (b) GFP expression in the central caudal nidopallium. (c) Co-localization of mCherry and GFP in the central caudal nidopallium. (d) mCherry expression in the hippocampus (e) GFP expression in the hippocampus. (f) Co-localization of mCherry and GFP in the hippocampus. Scale bars represent 200 μm.

Conditional expression using the Cre-loxP system offers precise control over transgene expression, making it particularly useful for optogenetic studies. In this approach, one vector expresses Cre recombinase, while a second vector contains a Cre-dependent promoter driving the transgene of interest. This ensures transgene expression only in the presence of Cre, enabling targeted manipulation of specific cells or projections (Tye and Deisseroth, 2012). To test this system, we injected AAV8-EF1a-dblf-mCherry alone or together with AAV8-CAG-Cre. In the absence of Cre, no mCherry expression was observed (Fig. 6a). However, co-injections with Cre resulted in high levels of mCherry expression (Fig. 6b,c,d,e), demonstrating the feasibility of this approach for optogenetic applications in pigeons. We verified this system for different brain areas including the hyperpallium (Fig. 6a and b), the mesopallium (Fig. 6a and b), the central caudal nidopallium (Fig. 7a–c), and the hippocampus (Fig. 7d–f).

## 3 Discussion

This study set out to investigate the usability and effectiveness of various viral vectors to expand the available toolkit for transgene delivery in pigeons, a species for which transgenic approaches are not yet feasible. Specifically, we tested AAV8 and AAVretro for their ability to transduce cells, examined the specificity of the CaMKIIα promoter and mDLX enhancer, and provided proof-of-concept for conditional systems such as Cre-loxP, laying the groundwork for future studies requiring precise gene transfer methods in this species.

### 3.1 AAV1 and AAV8 expression characteristics

AAV1 mediated transgene expression could be observed in the hyperpallium, entopallium, nidopallium, lateral striatum and olfactory bulb. This replicates and expands prior work with AAV1 demonstrating its usability in more areas of the pigeon brain (Rook et al., 2021). AAV8 was also able to drive transgene expression across multiple brain regions in pigeons, including the nidopallium, mesopallium, hyperpallium, hippocampus, striatum, and septum, without inducing neurotoxic effects. In our study, GFAP staining for reactive gliosis and NeuN staining for neuronal integrity revealed no abnormalities at injection sites, indicating that AAV8 is well-tolerated in the pigeon brain. AAV1 was also not associated with neurotoxic effects, which has also been previously reported (Rook et al., 2021). In contrast, previous studies reported neurotoxicity with AAV9 in pigeons (Rook et al., 2021), characterized by increased gliosis and decreased neuronal viability, highlighting species-specific differences in AAV serotype compatibility. These findings are consistent with rodent studies, where AAV8 is generally non-toxic when appropriately dosed but may induce neurotoxicity under certain conditions. For example, McLeod et al. (2024) found that AAV8 applications led to a mild inflammatory response, with a higher AAV titer correlating to more GFAP reactivity (McLeod et al., 2024). However, this inflammatory response did not result in a significant loss of neuronal tissue. Promoter choice also influences toxicity; ubiquitous strong promoters like CAG or CMV can exacerbate adverse effects, while cell-type-specific promoters may reduce toxicity by limiting transgene expression to targeted populations (Xiong et al., 2019). The absence of neurotoxicity in our study suggests that AAV8 offers a safer and more effective alternative to AAV9 for gene delivery in pigeons. This aligns with its favorable safety profile in rodents when optimized, reinforcing the need for species-specific testing to account for variations in vector performance and biocompatibility across models.

Moreover, we observed extensive anterograde transgene expression with labeling detected in regions distal to the injection sites, such as the arcopallium and midbrain structures. Retrograde transgene expression was also evident, although it appeared less pronounced than reported for AAV1 in earlier studies (Rook et al., 2021). While some reports in rodents have noted that AAV8 exhibits weaker retrograde transgene expression compared to serotypes like AAV1 and AAV9 (Castle et al., 2014; Cearley and Wolfe, 2006; Wang and Zhang, 2021), the degree of retrograde transgene expression in our study is difficult to compare directly due to differences in target areas and experimental protocols. Our study used multiple injections, which may have obscured retrograde transport from specific sites. In contrast, AAVretro showed limited effectiveness in pigeons, with only sparse transgene expression observed in a small number of neurons and negligible retrograde labeling. While AAVretro was specifically engineered for retrograde transport and performs robustly in rodent models (Tervo et al., 2016; Wang and Zhang, 2021), it appears to lack utility in pigeons, potentially due to species-specific differences in neuronal architecture or viral tropism. On the other hand, AAV1 and AAV8 demonstrate strong anterograde and retrograde capabilities in pigeons, making them more suitable for experiments requiring retrograde labeling or distal transgene expression.

Interestingly, AAV8 has been reported to transduce non-neuronal cells, such as oligodendrocytes and astrocytes, more efficiently than other serotypes (Castle et al., 2016; Aschauer et al., 2013). Our analysis showed that approximately 82% of transduced cells were NeuN-positive neurons, indicating strong neuronal tropism. This percentage is only marginally lower than the 86% found for AAV1 in this study, and indicates a very strong tropism for neurons. This seems a little surprising given that AAV8 should be more efficient in also transducing non-neuronal cells. However, a similar neuronal tropism has been reported in other species for AAV8. For example, in marmosets, a study found up to 99% of cells transfected in the motor cortex, striatum or substantia nigra were neurons (Masamizu et al., 2011). Interestingly, Klein et al. (2008) demonstrated that purification methods significantly impact AAV8 tropism (Klein et al., 2008). Vectors purified with iodixanol showed a preference for neurons, while those purified with CsCl transduced astroglial cells more effectively. Our use of chloroform-purified vectors resulted in a primarily neuronal expression pattern, with a modest increase in transduction of non-neuronal cells compared to AAV1 (18% vs. 14%). Overall, both AAV1 and AAV8 are able to drive expression in multiple different regions of the pigeon brain. However, as the titers were not available for all used constructs (Table 1), we refrained from potentially misleading quantitative comparison of total expression levels for AAV1 and AAV8. While it has been shown that AAV1-mediated expression is sufficient for successful optogenetic manipulation of behavior (Rook et al., 2021), our current findings suggest that AAV8 may offer versatility for targeting neurons and non-neuronal cells in pigeons, depending on the desired application and the purification method employed.

**Table 1:** Injected viral constructs.

Construct	Manufacturer	Animals (n)	Hemispheres (n)	Injection volume	Expression time	Analysis	Staining method	Figure
AAV8-CAG-tdTomato (Titer n/a)	Herlitze Lab	3 (Sex: 2m, 1f; Ages: 2-4y)	5	5 μl	6 weeks	General expression pattern, neuronal tropism, anterograde/retrograde transport	Fluorescence anti-RFP, anti-NeuN	1, 3, 4
AAV1-CAG-tdTomato (Titer n/a)	Herlitze Lab	3 (Sex: 2m, 1f; Ages: 7-9y)	4	5 μl	6 weeks	General expression pattern, neuronal tropism, anterograde/retrograde transport	Fluorescence anti-RFP	2, 3, 4
AAVretro-CAG-ChR2 (Titer ≥7 × 10^12^ vg/mL)	Addgene	3 (Sex and age n/a)	6	5 μl	6 weeks	General expression pattern, anterograde/retrograde transport	DAB anti-ChR2	4
AAV1-CaMKIIα-eNpHR3.0-eYFP (Titer ≥5 × 10^12^ vg/mL)	Addgene	5 (Sex: 4m, 1f; Ages: 3-11y)	8	5 μl	6 weeks	General expression pattern, GABA and CaMKIIα tropism	DAB anti-YFP; fluorescence anti-YFP, anti-GABA, anti-CaMKIIα	5
AAV1-mDLX-ChR2-mCherry (Titer ≥7 × 10^12^ vg/mL)	Addgene	4 (Sex: 4m; Ages: 3-10y)	6	5 μl	6 weeks	General expression pattern, GABA and CaMKIIα tropism	Fluorescence anti-RFP, anti-ChR2, anti-GABA, anti-CaMKIIα	5
AAV8.CAG.Cre.P2A.eGFP.SV40.WPRE (Titer n/a)	Herlitze Lab	2 (Sex: 2m; Ages: 7-10y)	2	5 μl	6 weeks	General expression pattern, Cre-dependent expression	Fluorescence anti-YFP	6, 7
AAV8.EF1a.dbfl.mCherry.CW3SL (Titer n/a)	Herlitze Lab	2 (Sex: 2m; Ages: 7-10y)	4	5 μl	6 weeks	General expression pattern, Cre-dependent expression	Fluorescence anti-RFP	6, 7

### 3.2 Cell type specificity

Another objective of this study was to evaluate viral vectors for cell-type-specific expression in pigeons, as previous work has been limited to the use of the broadly expressing CAG and CMV promoters and the neuron-specific hSyn promoter (Rook et al., 2021; Nimpf et al., 2024). To expand the toolkit, we tested the specificity of the CaMKIIα promoter and the mDLX enhancer. The mDLX enhancer has demonstrated selectivity for GABAergic interneurons in multiple species, including mice, ferrets, gerbils, marmosets, and zebra finches (Dimidschstein et al., 2016; Spool et al., 2021). This specificity likely arises from its derivation from the distalless homeobox 5 and 6 (Dlx5/6) genes, which are highly conserved across vertebrate species and are predominantly expressed in telencephalic interneurons (Zerucha et al., 2000; Ghanem et al., 2003).

We assessed the tropism of AAV1-mDLX in the pigeon brain through counterstaining for CaMKIIα and GABA. AAV1-mDLX exhibited overall weak expression levels and the co-localization analysis revealed a low overlap with CaMKIIα-positive neurons (8%) and a moderate overlap with GABA-immunoreactive cells (59%). This specificity aligns with expectations for mDLX but falls short of the over 90% co-localization with GABAergic neurons reported in other species (Dimidschstein et al., 2016). A possible explanation for this discrepancy is the known difficulty in accurately detecting GABAergic neurons due to the rapid degradation of GABA and challenges with antibody binding during immunohistochemical staining (Zhao et al., 2013). Nevertheless, the minimal overlap with CaMKIIα-positive neurons, which far outnumber GABAergic neurons (Spool et al., 2021), supports the idea that mDLX maintains a preference for GABAergic neurons in pigeons.

However, the low overall expression levels achieved with AAV1-mDLX suggest that this enhancer may not be optimal for GABAergic-specific gene transfer in pigeons. A potential alternative is the glutamate decarboxylase 1 (GAD1) promoter, which was recently shown to drive GABAergic expression effectively in zebra finches. While the GAD1 promoter and mDLX enhancer produced comparable results in zebra finches, testing the GAD1 promoter in pigeons could provide a more reliable method for targeting GABAergic neurons (Spool et al., 2021).

We also evaluated the CaMKIIα promoter, which is widely used for targeting excitatory glutamatergic neurons. In our study, the CaMKIIα promoter drove abundant expression of NpHR across pigeon forebrain regions, showing a strong preference for CaMKIIα-positive cells. However, this specificity was not absolute, as some co-localization with GABAergic neurons was observed. While the CaMKIIα promoter has been considered selective for excitatory neurons, recent studies have raised concerns about its exclusivity (Watakabe et al., 2015; Keaveney et al., 2020; Veres et al., 2023). Veres et al. (2023) demonstrated that the CaMKIIα promoter could drive expression in specific subclasses of GABAergic neurons, even in cases where immunohistochemical staining failed to detect CaMKIIα. Moreover, functional evidence showed that these neurons could be activated when expressing ChR2, despite low fluorescent signal levels in histological analysis. This finding is surprising since GABAergic neurons typically lack detectable CaMKIIα expression (Liu and Jones, 1996; Sík et al., 1998). One hypothesis is that the yCaMKII isoform, which is present in inhibitory neurons, may interact with the CaMKIIα promoter to facilitate gene expression (He et al., 2021).

Our results reflect this trend, as we observed some overlap with GABAergic neurons. However, the high specificity for CaMKIIα-positive neurons makes this promoter a viable option for targeting excitatory neurons compared to broad promoters like CAG. These findings underscore the importance of carefully validating the specificity of promoters for their intended applications in avian models.

### 3.3 Conditional gene expression

The Cre-loxP system enables conditional and projection-specific gene expression (McLellan et al., 2017). This system requires two components: a viral vector encoding the Cre recombinase and another vector carrying a transgene flanked by loxP recognition sites. In our experiments, injections with only the double-floxed vector did not produce any detectable expression, confirming the necessity of Cre recombinase for activating the target gene. Injections that included both the Cre vector and the double-floxed vector led to abundant transgene expression, with clear colocalization of Cre and reporter proteins. While our results support the efficacy of the Cre-loxP system in pigeons, we acknowledge recent concerns about potential neurotoxic effects associated with AAV-mediated Cre expression. For example, Rezai Amin et al. (2019) observed significant cell death, reduced dopamine levels, and behavioral alterations following AAV-Cre injections into the substantia nigra of rodents (Rezai Amin et al., 2019). In our study, NeuN and GFAP staining patterns did not reveal any signs of neurotoxicity, but we recommend researchers remain vigilant and conduct thorough histological assessments when employing this system.

## 4 Conclusions and future directions

This study provides key insights into the usability and limitations of viral vectors and genetic tools for transgene delivery in pigeons. AAV8 emerged as a useable candidate with strong neuronal tropism and anterograde/retrograde expression capabilities, while AAVretro proved ineffective. Moreover, AAV1 and AAV8 remain reliable options for projection specific experiments.

The CaMKIIα promoter demonstrated high specificity for excitatory neurons, making it suitable for targeting glutamatergic cells, though some expression in GABAergic neurons suggests caution in systems requiring complete exclusivity. In contrast, the mDLX enhancer showed limited expression, restricting its utility for behavioral studies. Future research should explore alternatives like the GAD1 promoter for improved targeting of inhibitory neurons. Proof-of-concept demonstrations of the Cre-loxP system confirm its potential for conditional gene expression in pigeons.

All in all, these tools expand the genetic toolkit for pigeons and lay the groundwork for advanced research in avian neuroscience.

## 5 Methods

### 5.1 Subjects

For this study 19 adult homing pigeons (*Columba livia*) were sourced from local breeders. The animals were individually housed, with food and water ad libitum, and subjected to a 12-h light-dark cycle.

The experiments were carried out in compliance with the European Communities Council Directive of September 22 2010 (2010/63/EU) and the specifications of the German law for the prevention of cruelty to animals and was approved by the animal ethics committee of the Landesamt für Natur, Umwelt und Verbraucherschutz (LANUV) NRW, Germany. We confirm that all methods were carried out in accordance with relevant guidelines and regulations and that the study was conducted in compliance with the ARRIVE guidelines.

### 5.2 Viral vector production

Recombinant AAV stocks were generated using the triple-transfection technique and purified with chloroform (Grieger et al., 2006; Eickelbeck et al., 2019). Briefly, HEK293T cells were co-transfected with the vector plasmid, serotype plasmid, and a helper plasmid using polyethylenimine. Seventy-two hours post-transfection, the cells were collected by low-speed centrifugation, resuspended in lysis buffer (150 mM NaCl, 50 mM Tris-Cl, pH 8.5), and subjected to 5–7 freeze-thaw cycles. Following lysis, DNase I and MgCl_2_ were added, and the mixture was incubated at 37 °C for 30 min. Polyethylene glycol (PEG-8000) was added to the supernatant to a final concentration of 10% (w/v), and the solution was incubated at 4 °C for 2 h. After centrifugation (3700×*g*, 4 °C, 20 min), the resulting PEG-precipitated pellet was resuspended in the clarified lysate.

For further purification, the resuspension was treated with additional PEG-8000, incubated at 4 °C for at least 1 h, and centrifuged again (3700×*g*, 4 °C, 20 min). The resulting pellet was dissolved in 50 mM HEPES buffer. Chloroform was then added in equal volume, the mixture was vortexed, and phase separation was achieved by centrifugation at room temperature (370×*g*, 5 min). The aqueous layer was recovered, filtered through a 0.22 μm syringe filter, and further concentrated using PEG-8000. The final AAV preparation was resuspended in PBS containing 0.001% pluronic F68, aliquoted, and stored at −80 °C.

### 5.3 Viral vector injections

The injections of the viral vectors were applied locally to the brain regions of interest (the number of injections for each vector can be found in Table 1). For the surgical procedures, anaesthesia was initiated with a combination of ketamine (Ketavet ®, Zoetis Deutschland GmbH, Germany, 60 mg/kg bodyweight IM), xylazine (Rompun ®, Bayer AG, Germany, 2 mg/kg body weight IM), and buprenorphine (Buprenovet ®, VetViva Richter GmbH, Austria, 0.5 mg/kg body weight IM) (Serir et al., 2024). During the operation, gas anaesthesia using isoflurane (Forane 100%, Abbott GmbH & Co. KG, Wiesbaden, Germany) was applied additionally (Serir et al., 2024). Before the procedure, the feathers on the head were cut to reveal the skin. Once the animals showed no pain reflexes and surgical tolerance was achieved, the birds were placed into the stereotactic apparatus. After an incision into the skin to expose the skull, craniotomies were drilled to reveal the brain tissue. The positions of the craniotomies depended on the location of the brain area. After removing the dura mater, the needle was inserted into the brain to perform the injections in the target regions. All injections were performed using a Hamilton syringe. For injections, the Hamilton syringe was slowly lowered to the target region and injections were performed in a single location at a rate of 2.5 μl per 1 min. After injections were finished, the syringe stayed in place for further 10 min to allow the virus to properly diffuse into the tissue. The injection target coordinates for the different brain regions were A10.0/ML3.0/DV5.0 for the medial nidopallium, A9.0/ML5.5/DV4.0 for the entopallium, A12.0/ML5.5/DV3.5 for the anterior nidopallium, A7.5/ML1.0/DV1.0 for the hippocampus, A10.0/ML2.0/DV2.0 for the hyperpallium and A10.0/ML2.0/DV3.0 for the mesopallium.

After 6 weeks to allow for transgene expression, the animals were transcardially perfused. Once a deep anaesthesia was achieved using equithesin (0.45 ml per 100 g body weight), the perfusion was started by replacing the blood with a 0.9% sodium chloride (NaCl) solution, followed by fixation with 4° cold 4% paraformaldehyde (PFA) in 0.12M phosphate buffer (pH 7.4). Once the tissue was fixed, the brain was removed and stored in a postfix solution at 4° for 2 h (4 % PFA and 30 % sucrose in phosphate buffered saline (PBS)). Afterwards the brains were stored in a 30 % sucrose solution for at least 24 h to remove residual water for cryoprotection. For slicing, the brains were embedded in 15% gelatin/30% sucrose and again kept in postfix for 24 h. The brains were sliced using a freezing microtome (Leica, Wetzlar, Germany) into 40 μm thick coronal sections.

## 6 Immunohistochemistry

As previous work has shown that native fluorescence in pigeon tissue seems to significantly underestimate expression levels, all transgene expression was visualized using immunofluorescence amplification (Rook et al., 2021). Every tenth slice of a brain series was used and all stainings were carried out with free-floating sections. As different staining combinations were used (see Table 2), the following protocol describes the general staining procedure. In a first step, slices were rinsed in PBS for 2 × 5 min. Then, the slices were incubated in a 10 % blocking solution in PBS with 0.3% Triton-X-100 (PBST) for 30 min. Following the blocking step, the slices were transferred into a primary antibody solution (in PBST), where they remained for 72 h at 4 °C. After the primary incubation and further rinsing (3 × 10 min), the slices were then transferred into the solution containing the secondary antibodies (in PBST). This incubation step lasted for 1.5 h at room temperature. After a final rinsing step (3 × 10 min) the sections were mounted onto glass slides (Superfrost Plus, Thermo Scientific) and covered using Fluoromount-G (SouthernBiotech, Birmingham, USA).

**Table 2:** Antibody combinations used for different experiments.

Experiment	Primary antibody	Manufacturer and RRID	Secondary antibody	Manufacturer and RRID
**Assessment of AAV8 for Gene Transfer in Pigeon Brains**	rb anti-RFP 1:1000	Antibodies-Online Cat# ABIN129578, RRID:AB_10781500	gt anti-rabbit Alexa Fluor 594 1:500	Thermo Fisher Scientific Cat# A32740, RRID:AB_2762824
	rat anti-GFAP 1:1000	Innovative Research Cat#13-0300, RRID:AB_86543	gt anti-rat Alexa Fluor 405 1:500	Thermo Fisher Scientific Cat# A48261, RRID:AB_2890550
	ms anti-NeuN 1:500	Thermo Fisher Scientific Cat# MA5-33103, RRID:AB_2802653	gt anti-mouse Alexa Fluor 488 1:250	Thermo Fisher Scientific Cat# A-11001, RRID:AB_2534069
**Comparison of AAV Serotypes for Anterograde and Retrograde Transport**	ms anti-ChR2 1:1000	Progen Cat#651180, RRID:AB_2892521	biotinylated horse anti-mouse IgG 1:500	Vector Laboratories Cat# BA-2000, RRID:AB_2313581
	rb anti-RFP 1:1000	Antibodies-Online Cat# ABIN129578, RRID:AB_10781500	gt anti-rabbit Alexa Fluor 594 1:500	Thermo Fisher Scientific Cat# A32740, RRID:AB_2762824
**Validation of Cell-Type Specific Promoter and Enhancer Systems for Transgene Expression**	ms anti-CaMKIIα 1:1000	Abcam Cat# ab22609, RRID:AB_447192	dk anti-mouse Alexa Fluor 488 1:500	Thermo Fisher Scientific Cat# A-21202, RRID:AB_141607
			dk anti-mouse Alexa Fluor 594 1:500	Thermo Fisher Scientific Cat# A-21203, RRID:AB_2535789
	rb anti-GABA 1:1000	Thermo Fisher Scientific Cat# PA5-32241, RRID:AB_2549714	dk anti-rabbit Alexa Fluor 488 1:500	Thermo Fisher Scientific Cat# A-21206, RRID:AB_2535792
			dk anti-rabbit Alexa Fluor 594 1:500	Thermo Fisher Scientific Cat# A-21207, RRID:AB_141637
	gt anti-YFP 1:1000	SICGEN Cat# AB1166, RRID:AB_2895441	dk anti-goat Alexa Fluor 488 1:500	Thermo Fisher Scientific Cat# A-11055, RRID:AB_2534102
	rb anti-RFP 1:1000	Antibodies-Online Cat# ABIN129578, RRID:AB_10781500	dk anti-rabbit Alexa Fluor 594 1:500	Thermo Fisher Scientific Cat# A-21207, RRID:AB_141637
	ms anti-ChR2 1:1000	Progen Cat#651180, RRID:AB_2892521	dk anti-mouse Alexa Fluor 594 1:500	Thermo Fisher Scientific Cat# A-21203, RRID:AB_2535789
**Evaluation of Conditional and Advanced Expression Systems in Pigeons**	rb anti-RFP 1:1000	Antibodies-Online Cat# ABIN129578, RRID:AB_10781500	dk anti-rabbit Alexa Fluor 594 1:500	Thermo Fisher Scientific Cat# A-21207, RRID:AB_141637
	gt anti-YFP 1:1000	SICGEN Cat# AB1166, RRID:AB_2895441	dk anti-goat Alexa Fluor 488 1:500	Thermo Fisher Scientific Cat# A-11055, RRID:AB_2534102

**Fig. 8:**
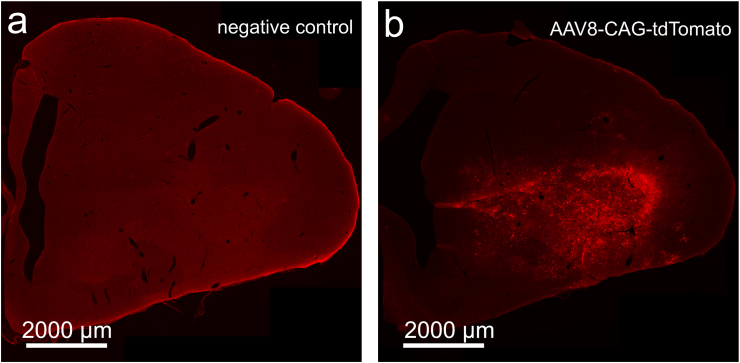
**Antibody specificity in immunohistochemical stainings against tdTomato.** (a) Staining result in a negative control slice without viral injection. (b) Staining result in a slice with AAV8-CAG-tdTomato injection. Scale bars indicate 2000 μm.

For DAB stainings, following the secondary antibody incubation and additional washing steps in PBS (3 × 10 min), the sections were then exposed to an avidin-biotin-peroxidase complex (1:100 in PBST, from the Vectastain Elite ABC kit) for 1 h. Following additional PBS rinses (3 × 10 min), the sections were placed in a DAB working solution. This solution consisted of 5 mL of distilled water mixed with 4 drops (100 μL) of DAB stock, 2 drops (84 μL) of buffer stock, and 2 drops (80 μL) of nickel solution. The sections were then transferred into individual cell wells, each containing 1 mL of the prepared DAB solution. To initiate the reaction, 3 μL of H_2_O_2_ solution was added to each well. After 2 min of incubation, the sections were moved to wells containing PBS and rinsed twice for 5 min each. Finally, the sections were mounted onto gelatin-coated slides, dehydrated through a graded alcohol series, and coverslipped using Depex (Fluka, Munich, Germany). We verified antibody specificity based on either published literature or stainings in negative control sections (Fig. 8). To verify reproducibility of our stainings, each staining was performed twice on separate brain series.

### 6.1 Microscopic analysis

The stained slices were imaged using the ZEISS AxioImager M1 and ZEISS AxioScan.Z1 at 100× magnification with an Orca Flash 4.0 V3 camera. All analyses were conducted manually using ZEN 3.5 lite. For the quantification of neuronal transduction for AAV1 and AAV8, regions of interest (ROIs) were manually selected within the entopallium and transgene expressing cells and the co-localization with NeuN were quantified. To determine the specificity of the CaMKIIα promoter ROIs were selected within the nidopallium and for the mDLX enhancer ROIs were selected within the entopallium. Transgene expressing cells and the respective co-localization with GABA and CaMKIIα were counted. Within the same images, the estimation of transduction efficiency for the AAV1-CaMKIIα and AAV1-mDLX constructs were manually assessed. Therefore, CaMKIIα was counted and the co-localization with AAV1-CaMKIIα-NpHR-eGFP transgene expression was quantified. For AAV1-mDLX, GABA was counted and the co-localization with RFP was quantified.

## Author contributions

N.R. conceived the experiments. T.S. and S.H. produced viral vectors. K.H. and J.M.T. conducted viral injections and perfusions. N.R., J.M.T., K.H., and M.Z. performed the histology and microscopic analysis. O.G. and S.H. acquired the funding. J.M.T. wrote the original draft of the paper. N.R. edited the paper. All authors reviewed the paper.

## Declaration of competing interest

The authors have nothing to declare.

## Data Availability

Data will be made available on request.
