# Supplementary material for: Achieving cell-type specific transduction with adeno-associated viral vectors in pigeons
Source: Curr Res Neurobiol. 2026 Apr 3;10:100157. doi: 10.1016/j.crneur.2026.100157 (PMC13091729; doi:10.1016/j.crneur.2026.100157)
Supplement: Multimedia component 1 [file mmc1.docx]

Peer Review Overview

**Manuscript Title:** Achieving cell-type specific transduction with adeno-associated

viral vectors in pigeons

| Received |  | Nov 06, 2025 |
| --- | --- | --- |
| 1st Decision |  | Jan 15, 2026 |
| 1st Revision Submitted |  | Feb 14, 2026 |
| 2nd Decision |  | Mar 20, 2026 |
| 2nd Revision Submitted |  | Mar 24, 2026 |
| Accepted |  | Mar 26, 2026 |

1st Decision letter

### **Reference:** CRNEUR-D-25-00017R2

### **Title:** Achieving cell-type specific transduction with adeno-associated viral vectors in pigeons

### **Journal:** Current Research in Neurobiology

Dear Dr. Rook,

Thank you for submitting your manuscript to Current Research in Neurobiology. 

I have completed my evaluation of your manuscript. The reviewers recommend reconsideration of your manuscript following major revision. I invite you to resubmit your manuscript after addressing the comments below. Please resubmit your revised manuscript by **Feb 14, 2026**. 

When revising your manuscript, please consider all issues mentioned in the reviewers' comments carefully: please outline every change made in response to their comments and provide suitable rebuttals for any comments not addressed. Please note that your revised submission may need to be re-reviewed.  

To submit a revision, go to https://www.editorialmanager.com/crneur/ and log in as an Author. You will see a menu item call Submission Needing Revision. You will find your submission record there.

When you submit the revised manuscript, please provide a separate document, uploaded as 'Detailed Response to Reviewers,' that carefully details, point-by-point, the list of changes made in response to the reviewers' comments. You may also include a suitable rebuttal to any specific request for change that you have not made. The journal strongly encourages you to submit two versions of your revised manuscript in order to facilitate the evaluation process: one with changes in the manuscript marked (upload as file type: Manuscript (REVISED, text with changes Marked)), in addition to an unmarked, production-ready version (required) (upload as file type: Manuscript).

Research Elements (optional)
This journal encourages you to share research objects - including your raw data, methods, protocols, software, hardware and more – which support your original research article in a Research Elements journal. Research Elements are open access, multidisciplinary, peer-reviewed journals which make the objects associated with your research more discoverable, trustworthy and promote replicability and reproducibility. As open access journals, there may be an Article Publishing Charge if your paper is accepted for publication. Find out more about the Research Elements journals at https://www.elsevier.com/authors/tools-and-resources/research-elements-journals?dgcid=ec_em_research_elements_email.


Current Research in Neurobiology values your contribution and I look forward to receiving your revised manuscript.

Kind regards,  

Anna S Mitchell, Ph.D. 
Editor in Chief 
Current Research in Neurobiology

**Comments from Editors and Reviewers:**

Your manuscript represents a very useful addition to the use of viral vectors in non-mammalian species. The reviewers have provided extensive supportive comments to improve clarity and details in your manuscript.

**Reviewer #1:**

This manuscript explores the use of AAVs to express molecular probes in the pigeon brain. Pigeons are an important model system in comparative neuroscience and are widely used to study the neural mechanisms underlying cognitive behaviors. A major gap in pigeon research and avian research generally is that many molecular tools used in systems neuroscience were developed and optimized for mice, and their efficacy and reliability in non-mammalian species remain uncertain. Therefore, the paper is important and of clear interest to the avian research community.
The results suggest that, among the AAV serotypes tested, AAV8 and AAV1 are effective vectors in pigeons. The study further indicates that these serotypes can support cell-type-specific expression of a range of molecular tools.
Overall, the methods and analyses are appropriate, and the results are largely convincing. However, several aspects could be improved, particularly the clarity of figure presentation and the way results are described. In addition, some methodological details are missing. See comments below:

Major comments
1. The abstract leaves the impression of AAV8 being the most efficient tool for use in pigeons. However, the results are presented as an unclear mix of AAV8 and AAV1 (e.g., Fig. 4 shows only AAV1, Fig. 5 only AAV8, and Fig. 6 again only AAV1). This creates confusion about the main take-home message. What is missing is a clear synthesis of the differences between the two vectors and an explicit summary of what was demonstrated with each serotype.

2. In most figures, close-ups near injection sites are shown without sufficient indication of the precise brain location (Figs. 2, 3, 4, and 6). To help readers orient the data anatomically, the manuscript should include low-magnification images of the relevant brain sections with clear markings of injection and expression sites (and/or schematic slice drawings with atlas coordinates where possible).

3. The conclusion from Fig. 3 is that AAVretro does not work in pigeons, neither retrogradely nor anterogradely, while AAV8 and AAV1 do. However, AAV1 and AAV8 were tested with an RFP reporter, whereas AAVretro was tested with ChR2. This confound should be addressed. For a valid comparison, the same reporter cassette should be used across vectors .

4. Across multiple figures, quantification of positive results is missing. For example, Fig. 5 provides a nice visual demonstration of Cre-dependent expression in a single experiment, and the table indicates this experiment was repeated in four birds. However, the outcomes of the other experiments are not shown or summarized. The manuscript should include either quantitative summaries across all replicates, or representative images from all replicates.

5. In its current form, the Tet-off demonstration seems premature, particularly since tetracycline-induced suppression was not tested. I suggest removing this figure from the manuscript or, alternatively, strengthening it by directly testing tetracycline-dependent deactivation.

6. The Methods section is not sufficiently detailed, leaving important aspects unclear. For example: Injection protocol details are missing (injection rate, dwell times, pipette movement/withdrawal protocol, number of deposits per track, etc.). Sex and age of animals should be reported. The data analysis description is incomplete: how were ROIs selected (manual vs. automated)? Provide more detail on the automatic segmentation process, including the training model, parameters, and any validation/quality-control steps.

Specific comments about results

1. "Using this method, we first quantified AAV1-CAG-tdTomato expression, which yielded a mean transduced area of 22.05% ± 1.65 SEM." - It is unclear how this percentage was calculated. Percent of what reference area (ROI, section, nucleus boundaries)? How was "transduced area" defined/segmented (thresholding, manual tracing, automated pipeline), and what criteria were used to classify pixels/regions as positive?

2. "We found that 25.27% ± 6.65 SEM (n = 3 animals) of all cells within the injection site were transgene-positive (Fig. 2b-d)." Panels 2b-d do not appear to present this quantification as described.
AAV8 transduction is quantified using both percent area and number of cells, whereas AAV1 is quantified only by area. For a clean comparison between vectors, the quantification approach should be consistent across serotypes.

3. Figure 1 - It is not clear how the red regions in the slice schematics (left panels) correspond to the microscopy images on the right. Consider adding rectangles on the slice schematics showing the field(s) of view, and/or explicitly marking injection sites and the extent of expression. Scale bars are missing for the slice schematics.

4. Figure 2 (panels b-d and f-h) - The injection sites are not clearly indicated. Adding low-magnification section images or slice schematics with marked injection/expression sites would be useful. Panel e is described as being in the hippocampus, but the figure would benefit from an illustration of the injection site/location in the brain to support this claim.

5. Figure 4 - The panel order is confusing. In the first two rows, the reporter labelling appears on the right (panels a and d) and the cell-type labelling is in the center; in the third and fourth rows this arrangement is reversed.

Specific comments about Discussion

1. "Provided proof-of-concept for conditional systems such as Cre-loxP and Tet-On/Tet-Off."
Since no tetracycline manipulation was performed, the claim of a Tet system proof-of-concept is questionable.

2. "The absence of neurotoxicity in our study suggests that AAV8 offers a safer and more effective alternative to AAV9 for gene delivery in pigeons." Given that a substantial portion of the manuscript focuses on AAV1, it is expected to comment on neurotoxicity (or lack thereof) for AAV1 as well.

3. "Avian tissue features higher neuronal densities compared to the mammalian neocortex [41], potentially leading to a lower relative fraction of transduced neurons per volume even if the absolute transduction efficacy were comparable." This argument is not clear. Why would the fraction of transduced neurons depend on neuronal density?


**Reviewer #2:**

This is a very interesting study investigating the effectiveness of adeno-associated viruses (AAV1, AAV8 and AAVretro) for transgene delivery in pigeons. Tools to investigate brain physiology are limited in birds, which makes this study a valuable resource for the avian neuroscience community at large.

In a previous study Rook et al. have shown that AAV1 is an effective viral vector for transgene delivery in pigeons (Rook et al., 2021). In this new study, Tuff, Haselhuhn et al. show that AAV8 is also an effective, non-toxic, neuronal specific viral construct for transgene delivery in various telencephalic brain regions. They further demonstrate cell type specific expression of reporter genes using AAV1 and the CaMKIIα promoter for excitatory neurons and the mDLX enhancer for inhibitory neurons. This marks an important step towards targeting and manipulating specific cell types in the pigeon brain. Finally they provide proof of concept experiments for the conditional expression of transgenes using the Cre/Lox and Tet-off systems.

This study advances our understanding of the effectiveness of AAV variants as gene delivery tools in pigeons and constitutes an important step toward establishing state-of-the-art viral and genetic toolkits for avian neuroscience, thereby expanding the experimental accessibility of non-traditional model species. I therefore recommend this manuscript for publication after the authors address a limited number of comments, questions, and suggestions aimed at clarifying key points that will help researchers more readily adopt these techniques.

1) "We injected AAV8 into various brain regions and observed widespread expression in areas such as the hippocampus (Fig. 1a, b), septum (Fig. 1a, c), mesopallium (Fig. 1d, e), nidopallium (Fig. 1d, f), entopallium (Fig. 1d, f), and hyperpallium (Fig. 1g, h, i). We quantified transgene expression by measuring the proportion of transgene-positive area in the injection site. For this, we extracted standardized 500 x 500 μm ROIs in areas with transgene expression for both AAV1-CAG-tdTomato and AAV8-CAG-tdTomato to compare expression levels."
Were all of the above stated regions used for the quantification of transgene expression? Please clarify.

2) Figure 2a-d
Similar question to above, which regions were used for the analysis of neuronal transduction ratio in Figure 2a? What brain region is shown in Figure 2b-d?

3) Figure 2f-h
What brain region is shown in Figure 2f-h.

4) Figure 3
What brain regions are shown Figure 3b, d, e, g, h and i? I suggest to label every image with the corresponding brain region in all figures to make this more clear overall.

Why was DAB staining performed for AAVretro and not fluorescent staining?

5) Figure 4
Spelling mistake in Figure legend 4e. It should read: "Fluorescence staining against CaMKIIα."

What brain regions are shown in Figure 4a-l?

I suggest to switch Figure 4g and h and Figure 4j and k around so that it fits with the structure of Figure 4a-b and d-e, where the transgene that was introduced is shown in the first panel of each row.

6) Figure 6
It is unfortunate that the addition of tetracycline, to test for downregulation of transgene expression using the Tet-Off system, was not included in the experiments shown in this manuscript. While acknowledging that evaluating transgene expression using the TetBow technique is valuable in its own, the absence of experiments addressing this key aspect of the Tet-off system raises the question of whether the experiments in their current form should be included in this manuscript, as opposed to being reported separately once the necessary additions have been implemented. At a minimum, it would be helpful to understand why these experiments were not included in the present study.
In its current form the experiment focuses more on the use of the TetBow system. It would be helpful to include additional representative examples of transduced neurons demonstrating that individual cells exhibit distinct combinatorial expression patterns (i.e., different relative mixtures) across the three fluorescent channels.

7) Viral titers and stereotactic coordinates
Including viral titers (not just volumes) as well as the stereotactic coordinates of all injections would improve the clarity and reproducibility of the study.

8) Sex of the animals used in the study
Including the sex of the animals used in the study would improve clarity and reproducibility.

1st Author Response Letter

**Response to comments from Editors and Reviewers:**

Thank you very much for your feedback and for the reviewers’ comments. We appreciate the

opportunity to revise our manuscript and have addressed all points raised. Below, we provide

a detailed response to each comment and describe the changes made in the manuscript. We

believe reviewers thorough examination of our manuscript has greatly improved the focus and

clarity of our paper.

Editor and Reviewer comments:

Your manuscript represents a very useful addition to the use of viral vectors in nonmammalian species. The reviewers have provided extensive supportive comments to improve

clarity and details in your manuscript.

Reviewer 1:

This manuscript explores the use of AAVs to express molecular probes in the

pigeon brain. Pigeons are an important model system in comparative neuroscience and are

widely used to study the neural mechanisms underlying cognitive behaviors. A major gap in

pigeon research and avian research generally is that many molecular tools used in systems

neuroscience were developed and optimized for mice, and their efficacy and reliability in non

mammalian species remain uncertain. Therefore, the paper is important and of clear interest to

the avian research community.

The results suggest that, among the AAV serotypes tested, AAV8 and AAV1 are effective

vectors in pigeons. The study further indicates that these serotypes can support cell-type

specific expression of a range of molecular tools.

Overall, the methods and analyses are appropriate, and the results are largely convincing.

However, several aspects could be improved, particularly the clarity of figure presentation

and the way results are described. In addition, some methodological details are missing. See

comments below:

Major comments

Comment 1: The abstract leaves the impression of AAV8 being the most efficient tool for use

in pigeons. However, the results are presented as an unclear mix of AAV8 and AAV1 (e.g., Fig.

4 shows only AAV1, Fig. 5 only AAV8, and Fig. 6 again only AAV1). This creates confusion

about the main take-home message. What is missing is a clear synthesis of the differences

between the two vectors and an explicit summary of what was demonstrated with each serotype.

Answer 1:

We thank the reviewer for their supportive comments on our manuscript.

We agree that the previous version of the manuscript may have appeared somewhat

fragmented regarding the roles of AAV1 and AAV8, and we appreciate the opportunity to

clarify the overall structure and take-home message.

In the revised manuscript, we have reorganized the Results section to provide a clearer logical

progression:

- Figures 1 and 2 demonstrate that both AAV1 and AAV8 robustly transduce multiple regions of the pigeon brain. We have included additional AAV1 data to present both serotypes in parallel and to improve clarity regarding their regional expression patterns.
- Figure 3 now presents the neuronal tropism (neuron-to-glia ratio) and assessment of potential neurotoxicity for both AAV1 and AAV8. The data show that both serotypes exhibit a strong neuronal preference and do not induce detectable gliosis at the injection site under our experimental conditions.
- Figure 4 illustrates that both AAV1 and AAV8 display anterograde and retrograde transport properties in the pigeon brain.

Together, these results demonstrate that AAV1 and AAV8 both function reliably in pigeons

with respect to transgene expression, neuronal tropism, transport characteristics, and safety

profile.

Importantly, Figures 5,6 and 7 shift the focus from serotype evaluation to the functionality of

promoter and conditional expression systems (CaMKIIα, mDLX, Cre-loxP). The preceding

figures establish that both AAV1 and AAV8 can serve as effective delivery vehicles in the

pigeon brain. The later figures therefore concentrate on validating genetic control strategies

rather than comparing serotypes.

We have revised the Abstract and Discussion to provide a clearer synthesis of what was

demonstrated with each serotype and to better highlight the overall take-home message of the

study.

Comment 2: In most figures, close-ups near injection sites are shown without sufficient

indication of the precise brain location (Figs. 2, 3, 4, and 6). To help readers orient the data

anatomically, the manuscript should include low-magnification images of the relevant brain

sections with clear markings of injection and expression sites (and/or schematic slice

drawings with atlas coordinates where possible).

Answer 2:

We agree that anatomical orientation is essential for proper interpretation of the data. In the

revised manuscript, we have addressed this concern by including schematic drawings of the

injection sites in all relevant figures. These schematics indicate the precise anatomical

location of the injections and corresponding expression areas, thereby providing clearer

spatial context for the higher-magnification images shown.

We believe that these additions substantially improve anatomical clarity and facilitate reader

orientation within the presented brain sections.

Comment 3: The conclusion from Fig. 3 is that AAVretro does not work in pigeons, neither

retrogradely nor anterogradely, while AAV8 and AAV1 do. However, AAV1 and AAV8

were tested with an RFP reporter, whereas AAVretro was tested with ChR2. This confound

should be addressed. For a valid comparison, the same reporter cassette should be used across

vectors.

Answer 3:

We agree that, ideally, all vectors should carry the same reporter cassette to allow for a fully

controlled comparison. In the present study, AAV1 and AAV8 were tested using a tdTomato

reporter, whereas AAVretro was only commercially available with a ChR2-mCherry construct

and not with tdTomato. Unfortunately, an AAVretro vector carrying an identical reporter

cassette was not available to us at the time of the experiments.

Importantly, we have previously validated AAV1 carrying ChR2-mCherry in pigeons (Rook

et al., 2021) and did not observe differences in transduction efficiency or expression patterns

compared to AAV1 carrying tdTomato. This suggests that the reporter cassette itself is

unlikely to account for the lack of detectable expression observed with AAVretro in the

present study.

Moreover, AAVretro was injected into a different brain region than AAV1 and AAV8.

Specifically, we targeted the entopallium–rotundus pathway, which had previously been

demonstrated to support both retrograde and anterograde transport using AAV1-ChR2

mCherry (Rook et al., 2021). By selecting this established pathway, we aimed to minimize the

possibility that a failure of retrograde transport could be attributed to the insert rather than to

the capsid properties of AAVretro.

The new figure looks as follows:

Figure 4. Anterograde and retrograde properties of AAV1, AAV8 and AAVretro. (a) Schematic image

indicating the injection site of AAV8. (b) RFP-labeled fibers and cell bodies observed outside the injection site, indicating successful anterograde and retrograde expression of the transgene. (c) Higher magnification image of b. RFP-positive cells identified in regions distal to the injection site, demonstrating retrograde transgene expression. (d) Higher magnification image of b. RFP-labeled fibers in the arcopallium, demonstrating anterograde transgene expression. (e) Schematic image indicating the injection site for AAV1. (f) RFP-labeled fibers and cell bodies observed outside the injection site, indicating successful anterograde and retrograde expression of the transgene. (g) Higher magnification image of f. RFP-positive cells identified in regions distal to the injection site, demonstrating retrograde transgene expression. (h) Higher magnification image of h. RFP-labeled fibers in the arcopallium, demonstrating anterograde transgene expression. (i) Schematic image indicating the injection site for

AAVretro. (j) Representative image of ChR2 expression at the injection site following AAVretro delivery,

highlighting local transduction efficiency. No ChR2-labeled fibers were observed outside the injection site, indicating unsuccessful anterograde transgene expression. (k) Virtually no ChR2-positive cells could be identified in regions distal to the injection site such as the nucleus rotundus, demonstrating virtually no retrograde transgene expression. The rounded area displaying a darker background corresponds to the nucleus rotundus, a structure characterized by naturally high signal intensity in DAB staining protocols. This is not indicative of non-specific labeling or an experimental artifact. (l) Higher magnification image of j depicting the injection site. The injected constructs were (a-d) AAV8-CAG-tdTomato (five hemispheres in three animals), (e-h) AAV1-CAG-tdTomato (five hemispheres in three animals) and (i-l) AAVretro (six hemispheres in three animals). Scale bars indicate (b, f, j, k, l) 500 µm, (c, d) 100 µm or (g, h) 200 µm.

Comment 4: Across multiple figures, quantification of positive results is missing. For

example, Fig. 5 provides a nice visual demonstration of Cre-dependent expression in a single

experiment, and the table indicates this experiment was repeated in four birds. However, the

outcomes of the other experiments are not shown or summarized. The manuscript should

include either quantitative summaries across all replicates, or representative images from all

replicates.

Answer 4:

We agree that a clearer summary of results across experimental replicates improves

transparency and interpretability. In the revised manuscript, we have therefore expanded the

presentation of the data as follows:

- For the promoter specificity experiments (CaMKIIα and GABA promoters), we now

include quantitative analyses of specificity, which are presented graphically in the

revised figures. These quantifications summarize results across all replicates and

provide a clearer overview beyond the originally shown representative images.

- Moreover, we have included the Neuron to Glia ratio also for AAV1 in Figure 3

(former Figure 2)

- For the Cre-dependent expression experiments, we now include images from the

additional biological replicates in Fig 6.

The new Figure looks as follows:

Figure 6. Conditional transgene expression using the Cre-LoxP system. (a) mCherry expression in the

central caudal nidopallium (b) GFP expression in the central caudal nidopallium. (c) Co-localization of mCherry and GFP in the central caudal nidopallium. (d) mCherry expression in the hippocampus (e) GFP expression in the hippocampus. (f) Co-localization of mCherry and GFP in the hippocampus. Scale bars represent 200 µm.

The new result section reads as follows:

We verified this system for different brain areas including the hyperpallium (Fig 5 a,b), the

mesopallium (Figure 5 a,b), the central caudal nidopallium (Fig. 6 a – c), and the

hippocampus (Fig 6 d-f).

Comment 5. In its current form, the Tet-off demonstration seems premature, particularly

since tetracycline-induced suppression was not tested. I suggest removing this figure from the

manuscript or, alternatively, strengthening it by directly testing tetracycline-dependent

deactivation.

Answer 5:

We agree that, without directly testing tetracycline-induced suppression, the Tet-off

experiment remains incomplete and may appear premature in its current form. In response to

this concern, we have removed the corresponding figure and related data from the manuscript.

The manuscript has been revised accordingly, and the text has been adjusted to maintain a

clear and coherent narrative without this experiment.

Comment 6: The Methods section is not sufficiently detailed, leaving important aspects

unclear. For example: Injection protocol details are missing (injection rate, dwell times,

pipette movement/withdrawal protocol, number of deposits per track, etc.). Sex and age of

animals should be reported. The data analysis description is incomplete: how were ROIs

selected (manual vs. automated)? Provide more detail on the automatic segmentation process,

including the training model, parameters, and any validation/quality-control steps.

Answer 6:

We thank the reviewer for pointing this out and have added more details to the methods

section.

Comment 7: 1. "Using this method, we first quantified AAV1-CAG-tdTomato expression,

which yielded a mean transduced area of 22.05% ± 1.65 SEM." - It is unclear how this

percentage was calculated. Percent of what reference area (ROI, section, nucleus boundaries)?

How was "transduced area" defined/segmented (thresholding, manual tracing, automated

pipeline), and what criteria were used to classify pixels/regions as positive?

Answer 7:

Upon reconsideration, we agree that the description of the “transduced area” measurement

was not sufficiently detailed and that this metric may not represent the most informative or

robust parameter for evaluating viral performance. Moreover, our main goal was not to

directly compare the two viral vectors. We have therefore removed the direct quantifications

to not suggest that one virus outperforms the other.

In the revised manuscript, we have therefore removed the area-based analysis entirely.

Instead, we now focus more on expression sites, anterograde and retrograde transport and the

neuron to glia ratio.

These parameters provide a clearer assessment of viral tropism and functional targeting, and

are less dependent on sectioning variability or thresholding procedures than area

measurements.

Comment 8: 2. "We found that 25.27% ± 6.65 SEM (n = 3 animals) of all cells within the

injection site were transgene-positive (Fig. 2b-d)." Panels 2b-d do not appear to present this

quantification as described.

AAV8 transduction is quantified using both percent area and number of cells, whereas AAV1

is quantified only by area. For a clean comparison between vectors, the quantification

approach should be consistent across serotypes.

Answer 8:

As described in our answer to comment 7, we do not directly want to compare AAV1 and

AAV8 and have removed the transduction efficiency for AAV1 and AAV8 from the

manuscript. Instead we have provided qualitative descriptions (Figue 1 and 2) for the

transgene expression across different regions for both serotypes. Moreover, we included the

neuron to glia ratio for both serotypes.

The manuscript and corresponding figures have been revised accordingly.

Comment 9: 3. Figure 1 - It is not clear how the red regions in the slice schematics (left

panels) correspond to the microscopy images on the right. Consider adding rectangles on the

slice schematics showing the field(s) of view, and/or explicitly marking injection sites and the

extent of expression. Scale bars are missing for the slice schematics.

Answer 9:

In the revised Figure 1, (and new Figure 2) we have improved the anatomical correspondence

between the slice schematics and the microscopy images. Specifically, we have:

- Added rectangles in the slice schematics indicating the exact field(s) of view shown in the corresponding microscopy images.
- Rearranged the images that are shown so that more landmarks are visible in the microscopic images

We believe these changes substantially improve clarity and allow readers to more easily relate the overview schematics to the higher-magnification images.

Comment 10: 4. Figure 2 (panels b-d and f-h) - The injection sites are not clearly indicated.

Adding low-magnification section images or slice schematics with marked

injection/expression sites would be useful. Panel e is described as being in the hippocampus,

but the figure would benefit from an illustration of the injection site/location in the brain to

support this claim.

Answer 10: In the revised version of Figure 2 (now Figure 3), we have added the neuron-to

glia ratio for AAV1, as well as GFAP expression at the injection site for AAV1. All analyses

shown in this figure were conducted in the entopallium. Panels b–d and f–h are intended to

visualize the quantifications derived from panels a and e, respectively.

In contrast to other figures in the manuscript, the precise anatomical position is less critical

here, as the primary aim is to illustrate the quantitative assessment rather than to emphasize

stereotaxic localization. Nevertheless, we have now included the label “E” (entopallium) in all

relevant images and explicitly state this information in both the Methods section and the

figure legend to ensure clarity.

Importantly, the image shown from the hippocampus does not depict the injection site.

Instead, it serves as a control to demonstrate the validity of the GFAP staining procedure.

While no GFAP expression was detected in the entopallium (injection site), robust GFAP

labeling was observed in the hippocampus, confirming that the staining protocol was

effective.

The new figure looks as follows:

Figure 3. Neuronal specificity and neurotoxicity for AAV1 and AAV8. (a) Neuronal transduction ratio

observed for injections with AAV8-CAG. (b-d) Fluorescence images depicting (b) RFP expression, (c) NeuN expression and (d) co-localization of RFP and NeuN for AAV8. (e) Neuronal transduction ratio observed for injections with AAV1-CAG (f-h)Fluorescence images depicting (f) RFP expression, (g) NeuN expression and (h) co-localization of RFP and NeuN for AAV1. Fluorescence images depicting (i, h) GFAP expression in a positive control area (the hippocampus), (j, n) RFP expression, (k,o) an absence of GFAP in the injection site and (l, p) colocalization of RFP and GFAP for (j-l) AAV8 and (n-p) AAV1. Injections were performed in five hemispheres of three pigeons. The neuronal transduction ratio was determined in a total of 22 sections for AAV8 and 19 sections for AAV1. Scale bars represent (b-h) 50 µm, (i, m) 100 µm or (j-l, n-p) 200 µm.

Comment 11: 5. Figure 4 - The panel order is confusing. In the first two rows, the reporter

labelling appears on the right (panels a and d) and the cell-type labelling is in the center; in

the third and fourth rows this arrangement is reversed.

Answer 11: We agree that the ordering was confusing. We have rearrange this figure entirely

and also included the quantifications and schematics as recommended in previous comments.

The new figure looks as follows:

Figure 5. CaMKIIα and GABA specificity of CaMKIIα and mDLX promoter/enhancer systems.

Fluorescence images of sections after injection of either (a-h) AAV1-CaMKIIα- eNpHR3.0-EYFP (n = eight

hemispheres in five animals) or (i-p) AAV1-mDLX-ChR2-mCherry (n = six hemispheres in four animals). (a) Schematic illustration indicating the location of AAV1-CaMKIIα injection. (b) Expression of NpHR after virus injection. (c) Fluorescence staining against GABA. (d) Overlay of NpHR and GABA signals showing no colocalizations. (e) Schematic illustration indicating the location of AAV1-CaMKIIα injection. (f) Expression of NpHR after virus injection. (g) Fluorescence staining again CaMKIIα. (h) Overlay of NpHR and CaMKIIα showing co-localization of both signals. (i) Schematic illustration indicating the location of AAV1-mDLX injection. (j) Expression of ChR2 after virus injection. (k) Fluorescence staining against CaMKIIα. (l) Overlay of CaMKIIα and ChR2 signals showing no co-localization. (m) Schematic illustration indicating the location of AAV1-mDLX injection. (n) Expression of ChR2 after virus injection. (o) Fluorescence staining against GABA. (p) Overlay of ChR2 and GABA showing co-localization of both signals. (q) Expression efficiency of AAV1CaMKIIα and AAV1-mDLX in the injection site. (r) Expression specificity for both constructs with GABA and CaMKIIα. Transgene expression was visualized with counterstainings against (a-f) YFP or (g-l) RFP. All scale bars represent 100 µm.

Comment 12: 1. "Provided proof-of-concept for conditional systems such as Cre-loxP and

Tet-On/Tet-Off."

Since no tetracycline manipulation was performed, the claim of a Tet system proof-of-concept

is questionable.

Answer 12:

We agree that, in the absence of tetracycline-dependent manipulation, the claim of providing

proof-of-concept for a Tet-On/Tet-Off system is not sufficiently supported. As also addressed

in our response to Comment 5, we have removed the Tet system experiment from the

manuscript.

Accordingly, we have revised the relevant statements in the abstract and main text and no

longer claim proof-of-concept for the Tet system. The manuscript now focuses on the

validated Cre-loxP approach.

Comment 13: 2. "The absence of neurotoxicity in our study suggests that AAV8 offers a

safer and more effective alternative to AAV9 for gene delivery in pigeons." Given that a

substantial portion of the manuscript focuses on AAV1, it is expected to comment on

neurotoxicity (or lack thereof) for AAV1 as well.

Answer 13:

The absence of neurotoxicity for AAV1 had already been demonstrated in our previous study

(Rook et al., 2021). However, we agree that, given the prominent role of AAV1 in the present

manuscript, this information should also be explicitly addressed within this study to allow a

direct and self-contained comparison.

We have therefore included additional data in the revised manuscript demonstrating the lack

of detectable neurotoxicity for AAV1 under the conditions tested here. The corresponding

image and analysis are now included in the relevant Figure 3.

Furthermore, we have revised the text to clearly state that both AAV1 and AAV8 showed no

evidence of neurotoxicity in our experiments.

Comment 14: 3. "Avian tissue features higher neuronal densities compared to the

mammalian neocortex [41], potentially leading to a lower relative fraction of transduced

neurons per volume even if the absolute transduction efficacy were comparable." This

argument is not clear. Why would the fraction of transduced neurons depend on neuronal

density?

Answer 14:

In the revised manuscript, we have decided to remove potentially misleading comparisons of

AAV1 and AAV8. This argument has been removed from the manuscript. To still answer the

reviewers query, we agree that neuronal density per se does not affect the probability of

transduction on a per-cell basis. However, in our experiments viral delivery is constrained by

a fixed injection volume. Under these conditions, avian pallial tissue—which contains a

substantially higher number of neurons per unit volume compared to the mammalian

neocortex—presents a larger number of target cells competing for a limited number of viral

particles. Consequently, even if the intrinsic transduction efficiency of individual neurons

were comparable across species, the fraction of neurons transduced within a given volume

could be reduced in avian tissue due to a lower effective virus-to-neuron ratio.

Reviewer 2: This is a very interesting study investigating the effectiveness of adeno

associated viruses (AAV1, AAV8 and AAVretro) for transgene delivery in pigeons. Tools to

investigate brain physiology are limited in birds, which makes this study a valuable resource

for the avian neuroscience community at large.

In a previous study Rook et al. have shown that AAV1 is an effective viral vector for

transgene delivery in pigeons (Rook et al., 2021). In this new study, Tuff, Haselhuhn et al.

show that AAV8 is also an effective, non-toxic, neuronal specific viral construct for transgene

delivery in various telencephalic brain regions. They further demonstrate cell type specific

expression of reporter genes using AAV1 and the CaMKIIα promoter for excitatory neurons

and the mDLX enhancer for inhibitory neurons. This marks an important step towards

targeting and manipulating specific cell types in the pigeon brain. Finally they provide proof

of concept experiments for the conditional expression of transgenes using the Cre/Lox and

Tet-off systems.

This study advances our understanding of the effectiveness of AAV variants as gene delivery

tools in pigeons and constitutes an important step toward establishing state-of-the-art viral and

genetic toolkits for avian neuroscience, thereby expanding the experimental accessibility of

non-traditional model species. I therefore recommend this manuscript for publication after the

authors address a limited number of comments, questions, and suggestions aimed at clarifying

key points that will help researchers more readily adopt these techniques.

Comment 15: 1) "We injected AAV8 into various brain regions and observed widespread

expression in areas such as the hippocampus (Fig. 1a, b), septum (Fig. 1a, c), mesopallium

(Fig. 1d, e), nidopallium (Fig. 1d, f), entopallium (Fig. 1d, f), and hyperpallium (Fig. 1g, h, i).

We quantified transgene expression by measuring the proportion of transgene-positive area in

the injection site. For this, we extracted standardized 500 x 500 μm ROIs in areas with

transgene expression for both AAV1-CAG-tdTomato and AAV8-CAG-tdTomato to compare

expression levels."

Were all of the above stated regions used for the quantification of transgene expression?

Please clarify.

Answer 15:

We thank the reviewer for their thoughtful comments on our manuscript.

No, not all of the regions listed were used for the quantification of transgene expression.

Quantification was performed specifically in the entopallium. In the revised manuscript, we

have clarified this more clearly. We now include schematic representations to better illustrate

the analyzed regions. In addition, we removed some of the previously included quantifications

(e.g., transfection density).

Importantly, we have now also included the neuron-to-glia ratio for AAV1 as well as

transfection sites for AAV1 that we have observed. The neuron-to-glia ratio analysis was

performed in the entopallium, and the Methods section has been updated to explicitly specify

this.

We hope this clarification resolves the concern.

Comment 16: 2) Figure 2a-d

Similar question to above, which regions were used for the analysis of neuronal transduction

ratio in Figure 2a? What brain region is shown in Figure 2b-d?

Answer 16:

The analysis of neuronal transduction ratio in Figure 2 (now Figure 3) was also performed in

the entopallium. We have extended the new Figure 3 to include the transduction ratio and

potential neurotoxicity for AAV1 as well. All shown images (except for panels i and m, which

are hippocampus) are from the entopallium.

Comment 17: 3) Figure 2f-h

What brain region is shown in Figure 2f-h.

Answer 17:

For the neurotoxicity analysis, we focused also on the entopallium, which represents the

injection site of these cases, for both AAV1 and AAV8. In the revised manuscript, we have

now also included AAV1 in this analysis. This has been clarified in the revised text.

The panels previously shown as Figure 2f–h (now Figure 3j–l and Figure 3n–p in the revised

manuscript) depict the entopallium for AAV8 and AAV1, respectively.

Comment 18: 4) Figure 3

What brain regions are shown Figure 3b, d, e, g, h and i? I suggest to label every image with

the corresponding brain region in all figures to make this more clear overall.

Answer 18:

Figure 3 (now Figure 4 in the revised manuscript) has been completely redesigned to improve

clarity. We now provide schematic illustrations of the respective injection sites on the left side

of the figure. For AAV8 and AAV1, we show the same brain regions to compare anterograde

and retrograde transport. In addition, we included clearer overview images to make the

anatomical localization more transparent.

In response to the reviewer’s suggestion, we have now labeled each image with the

corresponding brain region throughout all figures to avoid ambiguity.

Comment 19: Why was DAB staining performed for AAVretro and not fluorescent staining?

Answer 19:

We initially assessed AAVretro expression using fluorescence microscopy. However, we did

not detect a convincing signal. To ensure that this negative result was not due to limited

fluorescence sensitivity, we subsequently performed DAB immunohistochemical staining.

Based on our previous experience (Rook et al., 2021), DAB staining substantially enhances

signal detection compared to direct fluorescence and allows for more sensitive visualization

of low-level expression. We therefore considered DAB staining to be the more reliable

method to conclusively determine whether the vector produced detectable expression.

Despite this increased sensitivity, we still did not observe convincing labeling, supporting our

conclusion that the AAVretro vector did not function appropriately under the conditions

tested.

Comment 20: 5) Figure 4

Spelling mistake in Figure legend 4e. It should read: "Fluorescence staining against

CaMKIIα."

What brain regions are shown in Figure 4a-l?

I suggest to switch Figure 4g and h and Figure 4j and k around so that it fits with the structure

of Figure 4a-b and d-e, where the transgene that was introduced is shown in the first panel of

each row.

Answer 20:

We have corrected the spelling mistake in the figure legend. It now correctly reads:

“Fluorescence staining against CaMKIIα.”

In addition, the former Figure 4 (now Figure 5 in the revised manuscript) has been

substantially revised. We have included quantifications that were previously described in the

text but not shown as figures clearly indicated the specific brain regions in which the

quantifications were performed and labeled all panels with the corresponding brain regions to

improve clarity.

Following your suggestion, we have also rearranged the panels (including switching the

respective images) so that, consistent with the structure of Figure 4a–b and d–e, the panel

showing the introduced transgene now appears first in each row. We believe this substantially

improves the logical flow and readability of the figure.

Comment 21: 6) Figure 6

It is unfortunate that the addition of tetracycline, to test for downregulation of transgene

expression using the Tet-Off system, was not included in the experiments shown in this

manuscript. While acknowledging that evaluating transgene expression using the TetBow

technique is valuable in its own, the absence of experiments addressing this key aspect of the

Tet-off system raises the question of whether the experiments in their current form should be

included in this manuscript, as opposed to being reported separately once the necessary

additions have been implemented. At a minimum, it would be helpful to understand why these

experiments were not included in the present study.

In its current form the experiment focuses more on the use of the TetBow system. It would be

helpful to include additional representative examples of transduced neurons demonstrating

that individual cells exhibit distinct combinatorial expression patterns (i.e., different relative

mixtures) across the three fluorescent channels.

Answer 21:

After careful consideration, we have decided to remove the Tet-Off/TetBow experiment from

the revised manuscript.

We agree that functional validation of transgene downregulation following tetracycline

administration represents a key aspect of the Tet-Off system. Since these experiments were

not included in the present study, we believe that keeping this section in its current,

incomplete form could be misleading and might distract from the main focus of the

manuscript. Rather than presenting a partial characterization, we consider it more appropriate

to perform a comprehensive evaluation of the Tet-Off system—including tetracycline

mediated suppression—in a future, dedicated study.

As a consequence, Figure 6 and the corresponding text have been removed from the revised

manuscript.

Comment 22: 7) Viral titers and stereotactic coordinates

Including viral titers (not just volumes) as well as the stereotactic coordinates of all injections

would improve the clarity and reproducibility of the study.

Answer 22:

We agree that reporting viral titers and stereotactic coordinates improves transparency.

Stereotactic coordinates for all injections have now been added to the Methods section.

For commercially obtained viral vectors, the genomic titers provided by the manufacturer are

now reported in the revised manuscript.

Several viral preparations, however, were produced in-house using the well-established triple

transfection and chloroform purification protocol routinely applied in the Herlitze laboratory

(see citations below). These studies employed the same production standards and vector

designs and demonstrated robust expression and functional utility in both cell culture and

brain tissue. Although precise genomic titers were not determined for these specific batches

and are therefore not available retrospectively, each preparation was functionally validated

prior to in vivo application by confirming robust transgene expression in HEK293T cells,

consistent with widely used validation practices in the field.

The primary goal of the present study was to assess general usability, tropism, transport

characteristics, and promoter specificity of viral vectors in the avian brain, rather than to

perform direct quantitative comparisons of transduction efficiency across serotypes or

preparations. All viral constructs produced clear and reproducible transgene expression at the

injection sites and exhibited expected cellular specificity or transport behavior.

To ensure transparency, we now explicitly state in the Methods section which viral

preparations have available titers and acknowledge the absence of quantified genomic titers

for certain in-house-produced vectors as a limitation of the study.

Citations:

Barzan, R., Bozkurt, B., Nejad, M. M., Süß, S. T., Surdin, T., Böke, H., ... & Jancke, D. (2024). Gain control of sensory input across polysynaptic circuitries in mouse visual cortex by a single G protein-coupled receptor type (5-HT2A). Nature Communications, 15(1), 8078.

Azimi, Z., Barzan, R., Spoida, K., Surdin, T., Wollenweber, P., Mark, M. D., ... & Jancke, D. (2020). Separable gain control of ongoing and evoked activity in the visual cortex by serotonergic input. Elife, 9, e53552.

Eickelbeck, D., Karapinar, R., Jack, A., Suess, S. T., Barzan, R., Azimi, Z., ... & Herlitze, S. (2019). CaMello-XR enables visualization and optogenetic control of Gq/11 signals and receptor trafficking in GPCR-specific domains. Communications biology, 2(1), 60.

Karapinar, R., Schwitalla, J. C., Eickelbeck, D., Pakusch, J., Mücher, B., Grömmke, M., ... & Herlitze, S. (2021). Reverse optogenetics of G protein signaling by zebrafish non-visual opsin Opn7b for synchronization of neuronal networks. Nature Communications, 12(1), 4488.

Comment 23: 8) Sex of the animals used in the study Including the sex of the animals used in the study would improve clarity and reproducibility.

Answer 23: In the revised version of the manuscript we now state the sex and age of the

animals for the specific experiments in table 1.

2nd Decision letter

### **Reference:** CRNEUR-D-25-00017R2

### **Title:** Achieving cell-type specific transduction with adeno-associated viral vectors in pigeons

### **Journal:** Current Research in Neurobiology

Dear Dr. Rook,  

Thank you for submitting your manuscript to Current Research in Neurobiology. 

I have completed my evaluation of your manuscript. The reviewers recommend reconsideration of your manuscript following minor revision and modification. I invite you to resubmit your manuscript after addressing the comments below. Please resubmit your revised manuscript by **Apr 03, 2026**.

When revising your manuscript, please consider all issues mentioned in the reviewers' comments carefully: please outline every change made in response to their comments and provide suitable rebuttals for any comments not addressed. Please note that your revised submission may need to be re-reviewed.  

To submit your revised manuscript, please log in as an author at https://www.editorialmanager.com/crneur/, and navigate to the "Submissions Needing Revision" folder under the Author Main Menu. 

When you submit the revised manuscript, please provide a separate document, uploaded as 'Detailed Response to Reviewers,' that carefully details, point-by-point, the list of changes made in response to the reviewers' comments. You may also include a suitable rebuttal to any specific request for change that you have not made. The journal strongly encourages you to submit two versions of your revised manuscript in order to facilitate the evaluation process: one with changes in the manuscript marked (upload as file type: Manuscript (REVISED, text with changes Marked)), in addition to an unmarked, production-ready version (required) (upload as file type: Manuscript).

Research Elements (optional)
This journal encourages you to share research objects - including your raw data, methods, protocols, software, hardware and more – which support your original research article in a Research Elements journal. Research Elements are open access, multidisciplinary, peer-reviewed journals which make the objects associated with your research more discoverable, trustworthy and promote replicability and reproducibility. As open access journals, there may be an Article Publishing Charge if your paper is accepted for publication. Find out more about the Research Elements journals at https://www.elsevier.com/authors/tools-and-resources/research-elements-journals?dgcid=ec_em_research_elements_email.


Current Research in Neurobiology values your contribution and I look forward to receiving your revised manuscript.

Kind regards, 

Anna S Mitchell, Ph.D. 
Editor in Chief 
Current Research in Neurobiology

**Comments from Editors and Reviewers:**

Please can you review the Figure 4 and legends to check accuracy - Reviewer 2 and I are confused about the correspondence between what is shown and what is written.
 
**Reviewer #1:**

The authors addressed my comments, and I think that the paper in its current form is ready for publication/


**Reviewer #2:**

In this revised version the authors have addressed the reviewer's comments and questions. The clarifications, additional information and analyses have improved the transparency of the methods employed and strengthened the reader's understanding of the data and it's interpretations.

Final minor clarifications:

1) Figure 4
If I am not mistaken the red shaded regions in Figure 4a, e and i show where expression was observable, not the exact injection site as indicated in the figure legend.

2) "AAV1 could be observed in the hyperpallium, entopallium, nidopallium, lateral striatum and
olfactory bulb. This replicates and expands prior work with AAV1 demonstrating its usability in
more areas of the pigeon brain [8]."
It's not AAV1 that is observed but rather AAV1 mediated transgene expression.

2nd Author Response Letter

**Response to comments from Editors and Reviewers:**

We thank the Editor and the Reviewers for the positive evaluation of our revised manuscript. We have changed the figure description of figure 4, as well as the sentence in the discussion according to the suggestion of reviewer 2.

**The new figure legend reads as follows:**

Figure 4. Anterograde and retrograde properties of AAV1, AAV8 and AAVretro. (a) Schematic image indicating the transduced area at the injection site of AAV8 in the medial nidopallium. (b) RFP-labeled fibers in the arcopallium and cell bodies in the NCL observed outside the injection site, indicating successful anterograde and retrograde expression of the transgene. (c) Higher magnification image of b. RFP-positive cells identified in the NCL, demonstrating retrograde transgene expression. (d) Higher magnification image of b. RFP-labeled fibers in the arcopallium, demonstrating anterograde transgene expression. (e) Schematic image indicating the transduced area at the injection site for AAV1 in the anterior nidopallium. (f) RFP-labeled fibers in the arcopallium and cell bodies in the NCL observed outside the injection site, indicating successful anterograde and retrograde expression of the transgene. (g) Higher magnification image of f. RFP-positive cells identified in the NCL, demonstrating retrograde transgene expression. (h) Higher magnification image of h. RFP-labeled fibers in the arcopallium, demonstrating anterograde transgene expression. (i) Schematic image indicating the transduced area at the injection site for AAVretro in the entopallium. (j) Representative image of ChR2 expression at the injection site following AAVretro delivery, highlighting local transduction efficiency. No ChR2-labeled fibers were observed outside the injection site, indicating unsuccessful anterograde transgene expression. (k) Virtually no ChR2-positive cells could be identified in regions distal to the injection site such as the nucleus rotundus, demonstrating virtually no retrograde transgene expression. The rounded area displaying a darker background corresponds to the nucleus rotundus, a structure characterized by naturally high signal intensity in DAB staining protocols. This is not indicative of non-specific labeling or an experimental artifact. (l) Higher magnification image of j depicting the injection site. The injected constructs were (a-d) AAV8-CAG-tdTomato (five hemispheres in three animals), (e-h) AAV1-CAG-tdTomato (five hemispheres in three animals) and (i-l) AAVretro (six hemispheres in three animals). Scale bars indicate (b, f, j, k, l) 500 µm, (c, d) 100 µm or (g, h) 200 µm.

**The new sentence in the discussion reads as follows:**

AAV1 mediated transgene expression could be observed in the hyperpallium, entopallium, nidopallium, lateral striatum and olfactory bulb. This replicates and expands prior work with AAV1 demonstrating its usability in more areas of the pigeon brain [8].

Accept Letter

Dear Dr. Rook,   
 
Thank you for submitting your manuscript to Current Research in Neurobiology.

I am pleased to inform you that your manuscript has been accepted for publication. Congratulations.
    
Your accepted manuscript will now be transferred to our production department. We will create a proof which you will be asked to check, and you will also be asked to complete a number of online forms required for publication. If we need additional information from you during the production process, we will contact you directly.

We appreciate and value your contribution to Current Research in Neurobiology. We regularly invite authors of recently published manuscript to participate in the peer review process. If you were not already part of the journal’s reviewer pool, you have now been added to it. We look forward to your continued participation in our journal, and we hope you will consider us again for future submissions.

*CRNEUR* aims to be a unique, community-led journal, as highlighted in the [Editorial Introduction](https://www.sciencedirect.com/science/article/pii/S2665945X21000012). As part of this vision, we will be regularly seeking input from the scientific community and encourage you and your co-authors to take the [survey](https://www.surveymonkey.co.uk/r/5LHWTML).

We would also like to invite you to take part in our CRNEUR Author [Question & Answer (Q&A)](https://www.elsevier.com/__data/promis_misc/QA_Authors.docx), which could get published alongside your article and help to promote it. We suspect you might have an interesting story of perseverance or team work that was required for the research study to complete, or a diversity of perspectives that you might share, as a way of inspiring others about neuroscience.

Kind regards,

Anna S Mitchell, Ph.D.  
Editor in Chief  
Current Research in Neurobiology  

*-------- End of Review Comments --------*
